# Negative binomial additive model for RNA-Seq data analysis

**DOI:** 10.1186/s12859-020-3506-x

**Published:** 2020-05-01

**Authors:** Xu Ren, Pei-Fen Kuan

**Affiliations:** Department of Applied Mathematics and Statistics, Stony Brook University, Stony Brook, 11794 NY USA

**Keywords:** Bayesian shrinkage, Differential expression analysis, Generalized additive model, RNA-Seq, Spline model

## Abstract

**Background:**

High-throughput sequencing experiments followed by differential expression analysis is a widely used approach for detecting genomic biomarkers. A fundamental step in differential expression analysis is to model the association between gene counts and covariates of interest. Existing models assume linear effect of covariates, which is restrictive and may not be sufficient for certain phenotypes.

**Results:**

We introduce NBAMSeq, a flexible statistical model based on the generalized additive model and allows for information sharing across genes in variance estimation. Specifically, we model the logarithm of mean gene counts as sums of smooth functions with the smoothing parameters and coefficients estimated simultaneously within a nested iterative method. The variance is estimated by the Bayesian shrinkage approach to fully exploit the information across all genes.

**Conclusions:**

Based on extensive simulations and case studies of RNA-Seq data, we show that NBAMSeq offers improved performance in detecting nonlinear effect and maintains equivalent performance in detecting linear effect compared to existing methods. The vignette and source code of NBAMSeq are available at http://bioconductor.org/packages/release/bioc/html/NBAMSeq.html.

## Background

### Introduction

In recent years, RNA-Seq experiments have become the state-of-the-art method for quantifying mRNAs levels by measuring gene expression digitally in biological samples. An RNA-Seq experiment usually starts with isolating RNA sequences from biological samples using the Illumina Genome Analyzer, a commonly used platform for high-throughput sequencing data. These mRNA sequences are reverse transcribed into cDNA fragments. To reduce the sequencing cost and increase the speed of reading the cDNA fragments (typically a few thousands bp), these fragments are sheared into short reads (50-450 bp). These reads are mapped back to the original reference genomes/transcriptomes and the number of read counts mapping to each gene/transcript region are computed. RNA-Seq experiments are usually summarized as a count table with each row representing a gene/transcript and each column representing a sample.

An important aspect of statistical inference in RNA-Seq data is the differential expression (DE) analysis, which is to perform statistical test on each gene to ascertain whether it is DE or not. Several methods have been developed for DE test using gene counts data, including DESeq2 [1], edgeR [2], which are based on negative binomial regression model; voom [3], which is based on empirical Bayes model and BBSeq [4], which is based on beta-binomial regression model. DESeq2 [1] performs DE analysis in a three-step procedure. The normalization factors for sequencing depth adjustment are first estimated using median-to-ratio method. Both the coefficients and the dispersion parameters in negative binomial distribution are estimated by the Bayesian shrinkage approach to effectively borrow the information across all genes. Finally, the Wald tests or the likelihood ratio tests are performed to identify DE genes. edgeR also uses the negative binomial distribution to model gene counts. It assumes that the variance of gene counts depends on two dispersion parameters, namely the negative binomial dispersion and the quasi-likelihood dispersion. The negative binomial dispersion is estimated by fitting a mean-dispersion trend across all genes whereas the quasi-likelihood dispersion are estimated by Bayesian shrinkage approach [5]. The DE statistical tests are conducted based on either the likelihood ratio tests [6, 7] or the quasi-likelihood F-tests [8, 9]. Both the DESeq2 and edgeR are widely used due in part to their shrinkage estimators which can improve the stability of DE test in a large range of RNA-Seq data or other technologies that generates read counts genomics data. voom [3] is an alternative tool for DE analysis. It estimates the mean-variance relationship of the log-counts non-parametrically, followed by the limma empirical Bayes analysis pipeline [10]. On the other hand, BBSeq [4] assumes that the count data follows a beta-binomial distribution where the beta distribution is parameterized in a way such that the variance accounts for over dispersion. The parameters are estimated by the maximum likelihood approach and DE analysis is based on either the Wald test or the likelihood ratio test. Both the negative binomial and beta-binomial regression models belong to the generalized linear model (GLM) class. GLM is popular owing to the ease of implementation and interpretation, by assuming the link function is a linear combination of covariates. However, as illustrated in the motivating dataset below, the linearity assumption is not always appropriate and nonlinear models for linking the phenotype to gene expression in RNA-Seq data may be important. Stricker et al. (2017) [11] proposed GenoGAM, which is a generalized additive model for ChIP-Seq data. However, GenoGAM was developed for a different purpose, i.e., the nonlinear component was to smooth the read count frequencies across genome, and not for relating a nonlinear association between the phenotype and read counts.

In this paper, we introduce NBAMSeq, a generalized additive model for RNA-Seq data. NBAMSeq brings together the negative binomial additive model [12] and information sharing across genes for variance estimation which enhances the power for detecting DE genes, since treating each gene independently suffers from power loss due to the high uncertainty of variance, especially when the sample size is small [1]. By borrowing information across genes, NBAMSeq is able to model the nonlinear association while improving the accuracy of variance estimation and shows significant gain in power for detecting DE genes when the nonlinear relationship exists.

### Motivating datasets

**Fig. 1:**
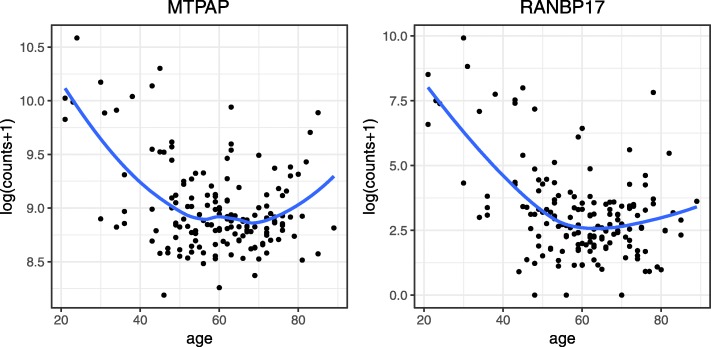
Nonlinear relationship between gene counts and age in TCGA GBM data

We start our exposition by demonstrating the existence of nonlinear relationship between gene counts and covariates/phenotypes of interest via three simple models and comparing them using ANOVA F-test. Two real RNA-Seq datasets are used. The first dataset is obtained from The Cancer Genome Atlas (TCGA) consortium, a rich repository consisting of high-throughput sequencing omics data for multiple types of cancers; downloadable from the Broad GDAC Firehose. For ease of exposition, we focus on the Glioblastoma multiforme (GBM), an aggressive form of brain cancer. The dataset consists of RNA-Seq count data on 20,501 genes and 158 samples generated using the Illumina HiSeq2000 platform. 16,092 genes were retained after filtering for genes with more than 10 samples having count per million (CPM) less than one. Gene counts are normalized using median-of-ratios as described in DESeq [13] and DESeq2 to account for different library sizes across all samples. Age has been identified as an important prognostic marker for malignant cancers including GBM [14]. To better understand the prognostic value of age in GBM, here we aim to identify age specific gene expression signature. For each individual gene *i*, three models were fitted: (i) base model: *y*_*ij*_=*β*_0_+*β*_1_*x*_1*j*_+*β*_2_*x*_2*j*_, (ii) linear model: *y*_*ij*_=*β*_0_+*β*_1_*x*_1*j*_+*β*_2_*x*_2*j*_+*β*_3_*x*_3*j*_, (iii) cubic spline regression with 3 knots: *y*_*ij*_=*β*_0_+*β*_1_*x*_1*j*_+*β*_2_*x*_2*j*_+*f*(*x*_3*j*_), where *y*_*ij*_ denotes logarithm transformed normalized counts of sample *j* and *x*_1*j*_,*x*_2*j*_ and *x*_3*j*_ denotes gender, race (Caucasian versus non-Caucasian) and age, respectively. We compared the three models using ANOVA F-test, and the *p*-values are adjusted using the Benjamini and Hochberg (1995) [15] false discovery rate (FDR). The test statistics, degrees of freedom in F-statistics and number of significant genes at FDR <0.05 are summarized in Additional file 1: Table S1. 250 genes are significant when comparing model (i) to model (iii). We further investigated the *p*-values of these 250 genes when comparing model (i) to model (ii) and found that 30 out of these 250 genes have FDR >0.2 (see Additional file 1: Table S2), implying that a simple linear regression is unable to identify these 30 genes even if a less stringent FDR threshold is used. Moreover, 9 genes are significant when comparing model (ii) to model (iii). Figure 1 shows the scatterplots of counts versus age of the most significant genes. For visualization, a local regression is fitted on each scatterplot, suggesting significant nonlinear relationships between normalized gene counts and age. Although only GBM is shown here, we observed similar nonlinear age effect for other types of cancer from Broad GDAC Firehose database (see Additional file 1: Table S3).

The second dataset comes from our study of gene expression associated with posttraumatic stress disorder (PTSD) in the World Trade Center (WTC) responders [16]. The dataset consists of RNA-Seq read counts of 25,830 genes in whole blood profiled in 324 WTC male responders. The phenotype of interest is the Posttraumatic Stress Disorder Checklist-Specific Version (PCL), a self-report questionnaire assessing the severity of WTC-related DSM-IV PTSD symptoms [17]. To better understand the nonlinear effect of PTSD severity on gene expression, here we aim to identify gene expression signature related to PCL score. The gene filtering criterion and normalization method are similar to the framework used in the first motivating dataset and samples with missing clinical information were omitted from the analysis. 16,193 genes and 321 samples were retained for DE analysis. In addition, cell type proportions have been implicated in the analysis of whole blood samples. The proportions of CD8T, CD4T, natural killer, B-cell and monocytes were estimated as described in our previous paper [18]. Similar to the first motivating dataset, the base model, linear model, and cubic spline regression model were fitted on each gene to study the effect of PCL score. Age, race, cell proportions of CD8T, CD4T, natural killer, B-cell and monocytes were adjusted in each model. When comparing the cubic spline regression model with base model using ANOVA F-test, 6 genes are significant at FDR <0.05. Additional file 1: Figure S1 shows the scatterplots of counts versus PCL score of these 6 genes. These plots show a consistent nonlinear pattern, i.e., gene expression first decrease and then gradually increase with the minimum at around PCL score 30.

These observations indicate that the relationship between gene counts and covariate of interest may be nonlinear, and it can be challenging to capture the true underlying nonlinear relationship using parametric methods. Thus, more flexible statistical methods to characterize the nonlinear pattern in RNA-Seq data will improve the DE analysis of complex phenotypes. Negative binomial distribution has been shown to be a powerful model because it captures the over-dispersion of the nature of the count data generated by RNA-Seq experiments [1, 2]. To this end, we propose a negative binomial additive model to capture the nonlinear association in RNA-Seq data analysis.

## Implementation

**Fig. 2:**
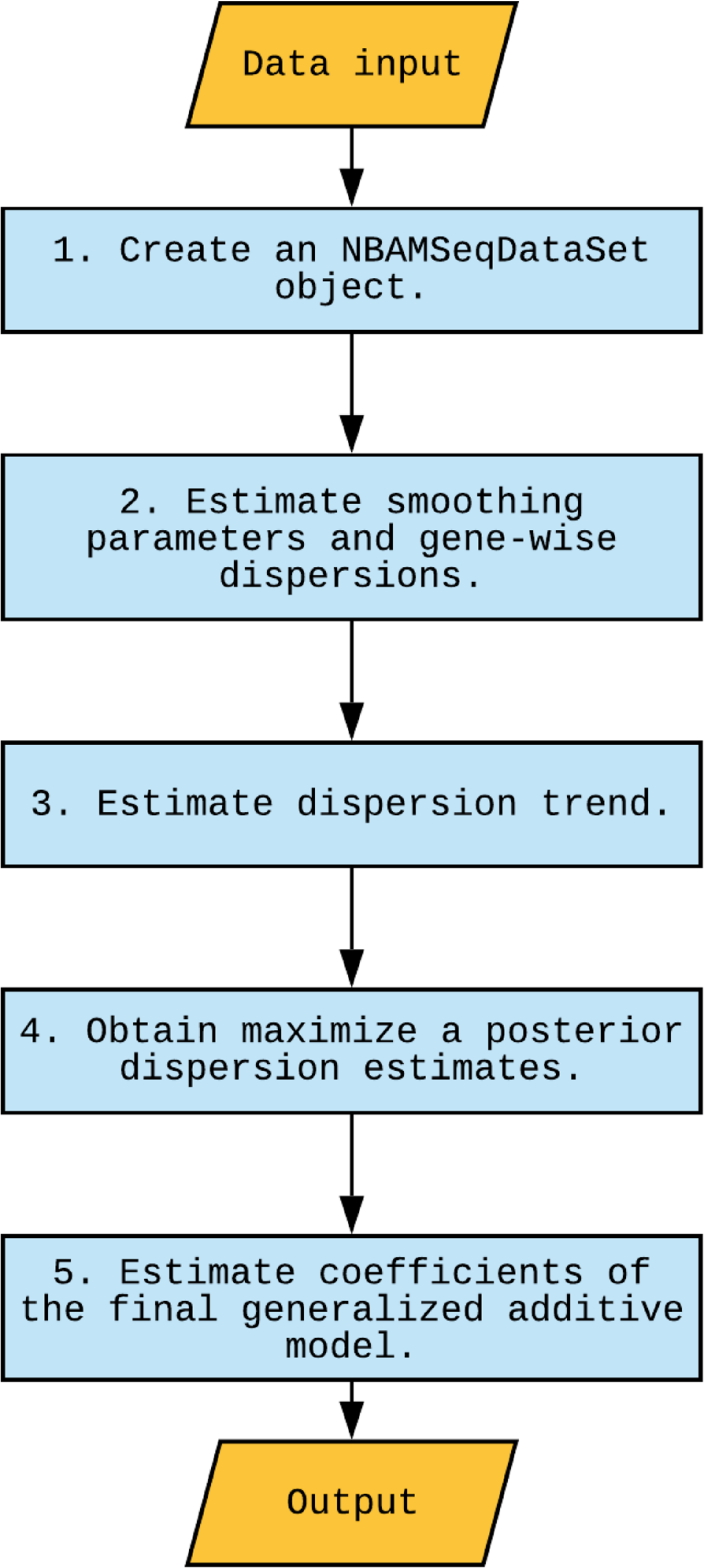
The workflow for NBAMSeq

NBAMSeq is implemented in R and its general work flow is summarized in Fig. 2. The foundations of generalized additive model, model fitting algorithm, parameter estimation, and statistical inference are given in Additional file 1 Information A. NBAMSeqDataSet is an R class used by NBAMSeq to store the count data and the estimated parameters during statistical analysis. To make NBAMSeq interoperable with other Bioconductor packages and facilitate downstream analysis of results, NBAMSeqDataSet is inherited from the Bioconductor core class SummarizedExperiment. Constructing a NBAMSeqDataSet object requires an RNASeq count matrix, a data frame which contains covariates of interest, and a design formula which specifies how to model the samples.

DE analysis is performed by NBAMSeq function which takes NBAMSeqDataSet objects as input. NBAMSeq function contains a few internal steps. The initial smoothing parameters, gene-wise dispersions, and coefficients of the covariates are estimated by gam function in mgcv [19–23]. These estimated values are saved in the NBAMSeqDataSet object to facilitate model fitting in the next steps. When using the gam function, to enforce smoother models we set the gamma argument to be 2.5 as the default value. The rationale of this default value is given in Additional file 1: Figure S4 and S5. The dispersion trend and maximum a posterior (MAP) dispersions are estimated by estimateDispersionsFit and estimateDispersionsMAP functions in DESeq2. The final coefficients are estimated by gam function again, where the smoothing parameters and initial values of coefficients are the previously saved values in the NBAMSeqDataSet object. To speed up these steps, parallel computing is supported by using parallel = TRUE option in the NBAMSeq function.

The DE test results can be obtained by the results function which takes the NBAMSeqDataSet after model fitting as input. The results can be visualized by the makeplot function.

## Results

### Simulation

To evaluate the performance of NBAMSeq, we designed simulation studies comparing NBAMSeq to DESeq2, edgeR and voom [3], three of the most popular softwares for identifying DE genes in RNA-Seq data. Four scenarios were considered; the first was to evaluate the accuracy of dispersion estimates and power of detecting DE genes when the nonlinear effect is the true association. The second was to investigate the robustness of NBAMSeq when the linear effect is the true association. The third was to evaluate the error rate control when no gene is differentially expressed, and the last was to investigate the ability of NBAMSeq in differentiating DE genes with nonlinear from linear effect.

In all scenarios, we assumed that the gene counts follow negative binomial distribution, i.e. *K*_*ij*_∼NB(*μ*_*ij*_,*α*_*i*_),*μ*_*ij*_=*s*_*j*_*q*_*ij*_. For simplicity and without loss of generality, we set all normalization factors *s*_*j*_ to be 1. Since *α*_*i*_ decreases with increasing mean counts, we assumed that the true mean dispersion relationship is $\alpha _{i} = a/ \bar {\mu }_{i}+ 0.05$. The performance under different degrees of dispersion (*a*=1,3,5) were studied. In practice, although *a* could be larger than 5, the larger value of *a* is compensated by the larger mean counts. The magnitude of *α*_*i*_ is in similar scale regardless of *a*, and thus only *a*=1,3,5 were studied. The covariate of interest *x*_*j*_ was simulated from a uniform distribution U(*u*_1_,*u*_2_). For simplicity, we used *u*_1_=20 and *u*_2_=80 because 20 to 80 covers the range of age and PCL score in our motivating datasets.

#### Scenario I

The aim of this scenario was two-fold: (i) to evaluate the accuracy of NBAMSeq dispersion estimates when nonlinear effect is significant, and (ii) to investigate the power and error rate control of NBAMSeq.

We simulated several count matrices which contain the same number of genes (*n*=15,000) across different sample sizes (*m* = 15, 20, 25, 30, 35, 40). We randomly selected 5% of the 15,000 genes to be DE. A comparison of other proportions of DE genes was given in Additional file 1: Figure S2 and S3, which shows that our proposed method is not sensitive to the DE proportions. Within each count matrix, the mean of negative binomial distribution *μ*_*ij*_ was simulated from

$$\eta_{ij} = \log_{2}(\mu_{ij}) = \left\{ \begin{array}{ll} b_{i0} + \sum_{q=1}^{5} b_{iq}B_{q}(x_{j}), & i \in \{\text{DE indices} \} \\ b_{i0} + c, & \text{otherwise} \end{array} \right. $$

and *μ*_*ij*_ was calculated by $\phantom {\dot {i}\!}\mu _{ij} = 2^{\eta _{ij}}$, where *B*(·) are cubic B-Spline basis functions to induce a nonlinear effect on DE genes, and *c* is a constant to ensure that the count distribution of DE and non-DE genes are comparable. The cubic B-Spline basis functions were generated by the bs function in spline package [24], and details of the recursive algorithm used by bs function can be found in Friedman et al. [25]. Since any nonlinear spline function can be generated from linear combination of B-Spline basis functions [26], we used the formula above to induce nonlinear effect in our simulations. The intercepts *b*_*i*0_ were simulated from normal distribution, and the other coefficients *b*_*i*1_,*b*_*i*2_,...,*b*_*i*5_ were simulated from a uniform distribution. The mean and standard deviation of normal distribution, as well as the lower/upper bound of uniform distribution were chosen such that the distribution of $\bar {\mu }_{i} = \frac {1} {m}\sum _{j = 1}^{m} \mu _{ij}$ across all genes mimicked our second motivating dataset. Finally, the count of gene *i* in sample *j* was simulated from NB(*μ*_*ij*_,*α*_*i*_). The simulation was repeated 50 times for each combination of dispersion and sample size. Without loss of generality, here we used the link function with base 2 in generating *μ*_*ij*_. One could also use link function with natural logarithm base in this simulation. Replacing base 2 with natural base only changes the coefficients of cubic B-Spline basis functions by a fixed constant, thus will not affect the accuracy of parameter estimation.

**Fig. 3:**
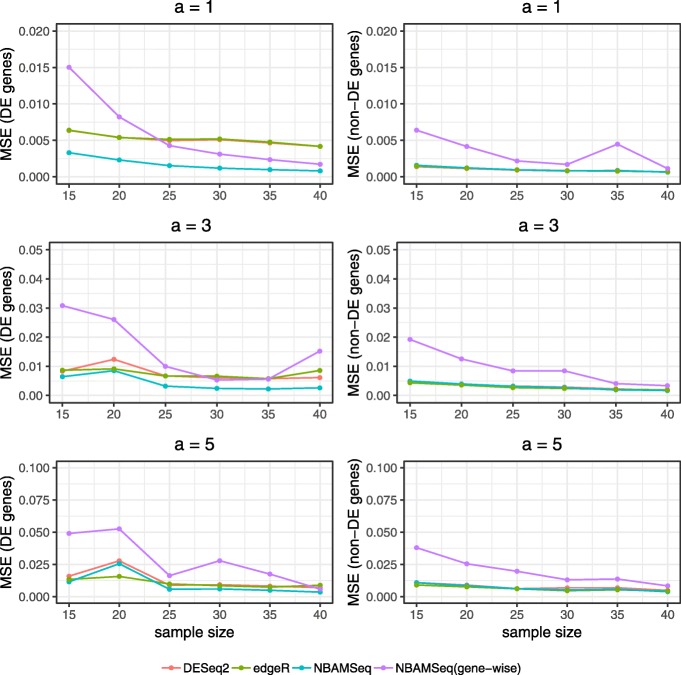
Scenario I: MSE of dispersion estimates

To evaluate the accuracy of dispersion estimates, three methods were compared: (a) DESeq2; (b) edgeR; (c) NBAMSeq. voom was excluded from dispersion estimates comparison because the model was applied on a different scale, i.e., log-counts based on linear model with Gaussian error, thus the notion of dispersion is not applicable for this method. The hypothesis of interest is whether the expression value of each gene is associated with the covariate of interest *x*_*j*_. For DESeq2 and edgeR, the design matrix is given by [***1***,**X**^∗^], where **X**^∗^=(*x*_1_,*x*_2_,...,*x*_*m*_)^T^. The hypothesis can be formulated as testing whether the coefficient of *x*_*j*_ in the generalized linear model is equal to zero. For NBAMSeq the design matrix is constructed by the method in Model Fitting section, and the hypothesis test are given by Inference section in Additional file 1: Information A. To illustrate that information sharing across genes produces more accurate dispersion estimates, the gene-wise dispersion estimated by NBAMSeq (NBAMSeq gene-wise) without incorporating prior dispersion trend was also included in the comparison. The average mean squared error (MSE) across 50 repetitions was calculated as shown in Fig. 3. Among the DE genes with nonlinear effect, NBAMSeq outperforms DESeq2 and edgeR across different sample sizes. On the other hand, among the non-DE genes, the MSE of edgeR, DESeq2 and NBAMSeq are comparable. NBAMSeq also has lower MSE than NBAMSeq (gene-wise), which shows that the Bayesian shrinkage estimation is better than gene-wise dispersion estimation. This simulation scenario shows that NBAMSeq is able to maintain good dispersion accuracy for non-DE genes and shows improvement in the accuracy over DESeq2 and edgeR for DE genes when the nonlinear association is significant.

**Fig. 4:**
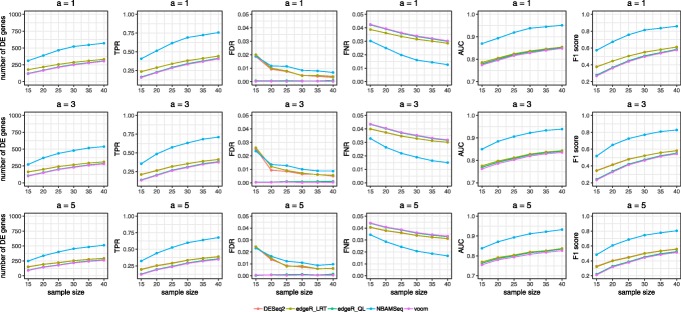
Performance metrics in Scenario I

Next, we compared the performance of DESeq2, edgeR, voom and NBAMSeq in DE analysis. The design matrix of voom is similar to DESeq2 and edgeR as given above. Figure 4 shows the average number of DE genes, true positive rate (TPR), empirical false discovery rate (FDR), empirical false non-discovery rate (FNR), area under curve (AUC) and F1 score of these four methods. For edgeR, two approaches for DE are available, namely the quasi-likelihood (QL) and the likelihood ratio test (LRT) approach for calculating the test statistics as described in Lund et al. (2012) [8]. The authors further showed that detecting DE genes using QL approach controls FDR better than the LRT approach. When calculating TPR, FDR, and FNR, genes are declared to be significant if nominal FDR is <0.05 (i.e. FDR adjusted p-value <0.05). The number of DE genes and TPR results indicate that our proposed method NBAMSeq has the highest power for detecting nonlinear DE genes regardless of the degrees of dispersion and sample sizes. In addition, NBAMSeq controls the FDR at 0.05 in all cases. The FNR results show that NBAMSeq has lower FNR compared to other methods. To ascertain that the power of NBAMSeq is not sensitive to the nominal FDR cutoff for DE genes, the AUC and F1 score comparisons show NBAMSeq is consistently more powerful in all cases. The observation also shows that the performance of DESeq2 and edgeR LRT approach are comparable, whereas edgeR QL approach is conservative as shown by the higher FNR. Additional comparisons of the QL approach for NBAMSeq and Gaussian additive models on logarithm transformed count data are provided in Additional file 1: Figure S4 and S5.

#### Scenario II

The goal of this scenario was to evaluate the robustness of NBAMSeq when the true association is a linear effect. The simulation setup in this scenario is similar to Scenario I except that *η*_*ij*_ is given by:

$$\eta_{ij} = \log_{2}(\mu_{ij}) = \left\{ \begin{array}{ll} b_{i1}x_{j} + c_{1}, & i \in \{\text{linear DE indices} \} \\ b_{i0} + c_{2}, & \text{otherwise} \end{array}\right. $$

where *c*_1_ and *c*_2_ are constants to ensure that the DE and non-DE genes counts distribution are comparable to avoid bias in detecting DE genes. The linear coefficient *b*_*i*1_ was simulated from normal distribution. Similar to Scenario I, the mean and standard deviation of normal distribution were chosen such that the distribution of $\bar {\mu }_{i}$ mimicked the distribution of mean normalized counts in real data. The simulation was repeated 50 times and the average MSE of dispersion estimates is shown in Additional file 1 Figure S6. The accuracy of NBAMSeq is comparable to the accuracy of DESeq2 and edgeR in almost all cases. This simulation shows that although NBAMSeq is designed to detect nonlinear effect, the method is robust and yields accurate dispersion estimates when the true association is linear.

To evaluate the power and error rate control of our proposed model NBAMSeq in detecting DE genes with linear effect, we compare the TPR, FDR, FNR, and AUC at nominal FDR 0.05. Additional file 1: Figure S7 illustrates that NBAMSeq is a robust approach and maintains comparable performance to other methods.

#### Scenario III

The aim of this scenario was to investigate the error rate control of NBAMSeq in the absence of DE genes. The simulation setup in this scenario is similar to Scenario I and II except that *η*_*ij*_ is given by *η*_*ij*_= log2(*μ*_*ij*_)=*b*_*i*0_+*c*. To evaluate the error rate control of the methods, we calculated the type I error. For each individual gene, the type I error is defined as the number of false positives over the total number of repetitions. Additional file 1 Figure S8 shows the box-plot of type I error across all genes. The type I error of NBAMSeq is comparable to that of other methods. The MSE plots of dispersion estimates are provided in Additional file 1: Figure S9, which shows the accuracy of dispersion estimates given by NBAMSeq is also comparable to the other methods.

#### Scenario IV

In real datasets, one could encounter scenarios in which both nonlinear and linear effect exist between gene counts and covariates. To mimic this situation, we considered a scenario where *μ*_*ij*_ was simulated from

$$\eta_{ij} = \log_{2}(\mu_{ij}) = \left\{ \begin{array}{ll} b_{i0} + \sum_{q=1}^{5} b_{iq}B_{q}(x_{j}), & i \in \{\text{nonlinear DE indices} \} \\ b_{i1}x_{j} + c_{1}, & i \in \{\text{linear DE indices} \} \\ b_{i0} + c_{2}, & \text{otherwise} \\ \end{array}\right. $$

A total of 5% genes was chosen to be DE genes, where 2.5% were nonlinear DE genes and the rest were linear DE genes. The median degree of dispersion (*a*=3) was studied. To determine whether a gene was differentially expressed or not, we used the same criteria as the previous scenarios, i.e., nominal FDR <0.05 to identify DE genes. To differentiate the two types of DE genes (linear versus nonlinear effect), we considered two approaches. In the first approach, a DE gene is declared to have nonlinear effect if its estimated effective degrees of freedom (edf) in generalized additive model [22] is above a certain threshold. Since a linear effect covariate only has one degree of freedom, we considered a DE gene as nonlinear if its edf >1.5. In the second approach, we differentiated the nonlinear and linear effect by comparing the AIC/BIC computed from the nonlinear and linear model. The AIC and BIC for nonlinear model was given in our NBAMSeq output, where the number of parameters in AIC/BIC was defined as edf [22]. We also fitted a linear model for each gene where the model fitting and parameter estimation procedure were similar to the “Implementation” section except that only linear terms were included in the model. A gene was considered to be nonlinear if its AIC/BIC in nonlinear model was lower than linear model. The correct classification percentage (CCP), which is defined as the total correctly classified genes over all genes, is given in Additional file 1: Table S4. The CCP for both approaches were high (>0.97 for all sample sizes) since majority genes were non-DE genes, therefore they contributed most to the CCP. Thus, we also computed the CCP for differentiating nonlinear and linear effect among the DE genes (Additional file 1: Table S5). The results showed the edf approach has higher accuracy compared to AIC/BIC approach. One possible explanation is that the generalized additive model automatically shrinks to a linear model when the true effect is linear. As a result, the AIC/BIC for linear and nonlinear models are very close, making the direct comparison difficult. In our software, both options, i.e., edf and AIC/BIC are provided. Based on our simulation results, we recommended the edf approach. For cases where the edf is large, the users may also use AIC/BIC for model comparisons.

### Real data analysis

**Fig. 5:**
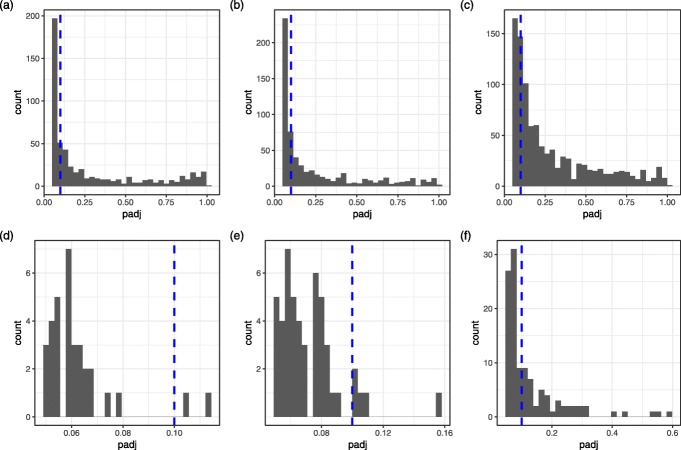
Histogram of adjusted p-values. The dashed line is FDR cut-off 0.1. **a** Genes detected by NBAMSeq but not DESeq2. **b** Genes detected by NBAMSeq but not edgeR. **c** Genes detected by NBAMSeq but not voom. **d** Genes detected by DESeq2 but not NBAMSeq. **e** Genes detected by edgeR but not NBAMSeq. **f** Genes detected by voom but not NBAMSeq

In this section, we applied DESeq2, edgeR with LRT approach, voom and our proposed method NBAMSeq to our motivating datasets (“Motivating datasets” section), namely the TCGA GBM dataset and WTC dataset. In TCGA GBM dataset, the association between age and each gene expression was investigated, adjusting for sex and race. At FDR 0.05, 845, 784, 510 and 1,349 genes were detected by DESeq2, edgeR, voom and our proposed NBAMSeq, respectively. 323 genes were in common among the four methods, whereas 440 genes were unique to NBAMSeq (Additional file 1: Figure S10). To account for the fact that significant genes are sensitive to the FDR threshold, we investigated the adjusted p-value of genes detected by NBAMSeq but not DESeq2, edgeR or voom and vice versa (see Fig. 5). A large proportion of the genes detected by NBAMSeq but not DESeq2, edgeR or voom have FDR above 0.1 for DESeq2, edgeR or voom, implying that these methods are unable to detect all the nonlinear genes even if a less stringent FDR threshold is used. However, for the genes detected by DESeq2, edgeR or voom but not NBAMSeq, the majority of their adjusted p-values in NBAMSeq are <0.1, implying that NBAMSeq is able to detect almost all these genes at FDR <0.1. Next, we performed the pathway analysis on DE genes using the hypergeometric tests implemented in the clusterProfiler software [27] for both the Kyoto Encyclopedia of Genes and Genomes (KEGG) [28] and Gene Ontology [29] gene sets. Significant pathways were chosen at FDR 0.05. Gene sets with fewer than 15 genes or greater than 500 genes were omitted from the analysis. For the KEGG pathways, no gene set was significant among DESeq2, edgeR, or voom DE genes whereas 1 genes set was significant among NBAMSeq DE genes (Table 1). This result is particularly interesting as cytokines dysregulation is involved in disrupting the immune system at older age via the loss of function to control systemic inflammation [30, 31], which could increase the susceptibility to age related diseases including cancer [32, 33]. In addition, the second gene set identified by NBAMSeq DE genes, i.e., Wnt signaling pathway (despite not making the FDR cutoff) was also an important pathway, as the mutations in Wnt signaling pathway is implicated in a variety of developmental defects in animals and associated with aging [34, 35]. For the Gene Ontology, a total of 5,000 gene sets were tested, 45, 12, 0 and 28 gene sets were identified by DESeq2, edgeR, voom and NBAMSeq, respectively and 23 gene sets were in common between DESeq2 and NBAMSeq. A full list of significant gene sets identified by these models are provided in Additional file 1: Table S6-S8. Specifically, the gene sets unique to NBAMSeq DE genes (Additional file 1: Table S9) are extracellular matrix pathways. Changes to extracellular matrix changes with aging has been implicated in the progression of cancer [36]. These observations suggest that gene sets detected by the NBAMSeq provide additional insights to the development of cancer and aging as a risk factor.

**Table 1:** Top 5 KEGG pathways selected by NBAMSeq

ID	Description	*p*value	p.adjust
hsa04060	Cytokine-cytokine receptor interaction	0.000154	0.0463
hsa04310	Wnt signaling pathway	0.001509	0.2271
hsa00260	Glycine, serine and threonine metabolism	0.003616	0.3628
hsa00350	Tyrosine metabolism	0.007484	0.5632
hsa04512	ECM-receptor interaction	0.010991	0.6555

In the WTC dataset, the relationship between PCL and gene expression were investigated, adjusting for age, race and cell proportions (CD8T, CD4T, natural killer, B-cell, monocytes). At FDR 0.05, 836, 770, 887 and 803 genes were detected by DESeq2, edgeR, voom and NBAMSeq, respectively (Additional file 1: Figure S11). Unlike the TCGA GBM dataset, the genes detected by the four methods have a large overlap, implying that the primary effect of PCL on gene expression is linear. Although NBAMSeq identified fewer PCL gene expression signature compared to DESeq2, the 44 genes detected by DESeq2 but not NBAMSeq were marginal significant in NBAMSeq (Additional file 1: Figure S12). However, for the genes unique to NBAMSeq, they were not necessarily significant in DESeq2, edgeR or voom. We performed pathway analysis on KEGG [28], and Additional file 1: Table S10 shows the significant KEGG pathways among NBAMSeq DE genes. As expected, these KEGG pathways were also significant among DESeq2 DE genes due to the large overlapping genes.

## Discussion

Although our model was developed for RNA-Seq data, it can be extended to identify nonlinear associations in other genomics biomarkers generated from high throughput sequencing which share similar features as RNA-Seq data (e.g., over dispersion in count data) as well as time-course RNA-Seq data. Compared to DESeq2, edgeR and voom, our proposed NBAMSeq method offers more flexibility as the degrees of freedom are estimated automatically within the model instead of being pre-specified. A closely related R package gam [37] is also developed for fitting generalized additive models. However, this package does not support negative binomial distribution and the degrees of freedom have be pre-specified in the spline function. Our NBAMSeq framework allows for flexible models to be fitted on individual genes via mgcv [19–23]. As shown in our simulation studies, the individual gene-wise dispersion estimate is less accurate compared to methods which model the information sharing across genes. Our proposed model is able to consider both the model flexibility for the non-linear relationships in the mean function as well as information sharing across genes in dispersion parameter estimation. In addition, NBAMSeq also offers the flexibility of differentiating the DE genes, i.e., linear versus nonlinear effect. In our simulation studies, we showed that the edf threshold 1.5 achieved good CCP, however future work includes determining the optimal edf threshold. Our simulation studies show that the proposed NBAMSeq is powerful when the underlying true association is nonlinear and robust when the underlying true association is linear compared to DESeq2, edgeR or voom. One might argue that the nonlinear association can be detected by fitting a spline regression with certain degrees of freedom and then incorporating the design matrix into DESeq2, edgeR or voom. The main disadvantage of this approach is that the degrees of freedom has to be fixed before using DESeq, edgeR or voom; and inappropriate choice of degrees of freedom leads to inflated FDR or underestimated TPR (see Additional file 1: Figure S13 - S15). In addition, the real data analysis showed that NBAMSeq offers additional biological insights to the role of aging in the development of cancer by modeling the nonlinear age effect. Both the simulation and case studies suggest the advantageous of investigating potential nonlinear associations to detect more effective biomarkers, in order to better understand the biological underpinnings of complex diseases.

NBAMSeq is developed for detecting nonlinear association between gene expression and continuous phenotypes. Our proposed framework is also applicable for scenarios in which the phenotype of interest is binary (e.g., cancer vs noncancer) and the model includes confounding variables which could be related to gene expression in a nonlinear pattern. On the other hand, for scenarios in which the phenotype of interest is ordinal, we recommend treating the variable as categorical if the number of categories is small (<5). If the number of categories is five or more, then one can treat it as a continuous variable and apply NBAMSeq for detecting nonlinear effect. Similar to other softwares for RNA-Seq DE analysis, currently NBAMSeq is developed for inference at gene level in the absence of prior information. Recent methods which take into account the gene network structure in DE analysis have been shown to a powerful approach [38]. Thus, an interesting future research direction is to incorporate the network topology in NBAMSeq to detect nonlinear association and decipher the collective dynamics and regulatory effects of gene expression. Currently, the computational time of NBAMSeq is longer compared to DESeq2, edgeR or voom due to the nested iteration algorithm. Future work includes approximating smoothing parameters by single iteration and reducing runtime cost.

## Conclusions

This paper introduced a flexible negative binomial additive model combined with Bayesian shrinkage dispersion estimates for RNA-Seq data (NBAMSeq). Motivated by the nonlinear effect of age in TCGA GBM data and PTSD severity (PCL score) in WTC data, our model aimed to detect genes which exhibit nonlinear association with the phenotype of interest. The smoothing parameters and coefficients in NBAMSeq were estimated efficiently by adopting the nested iterative method implemented in mgcv [19–23]. To increase the accuracy of the dispersion parameter estimation in small sample size scenario, the Bayesian shrinkage approach was applied to model the dispersion trend across all genes. Hypothesis tests to identify DE genes were based on chi-squared approximations. Finally, NBAMSeq is available as a Bioconductor package.

## Availability and requirements

**Project name:** NBAMSeq **Project home page:**http://bioconductor.org/packages/release/bioc/html/NBAMSeq.html**Operating system:** Platform independent **Programming language:** R **Other requirements:** R 3.6 or higher **License:** GPL-2 **Any restrictions to use by non-academics:** none

## Supplementary information

**Additional file 1** This file contains supplementary information, figures and tables.

## Abbreviations

AUC: Area under curve
CPM: Count per million
DE: Differentially expression
FDR: False discovery rate
FNR: False non-discovery rate
GBM: Glioblastoma multiforme
GLM: Generalized linear model
KEGG: Kyoto encyclopedia of genes and genomes
MAP: Maximize a posterior
MSE: Mean squared error
NBAMSeq: Negative binomial additive model for RNASeq dataset
PCL: PTSD Checklist-Specific Version
PTSD: Posttraumatic stress disorder
QL: Quasi-likelihood
TCGA: The cancer genome atlas
TPR: True positive rate
WTC: World trade center

## References

[1] Love MI, Huber W, Anders S (2014). Moderated estimation of fold change and dispersion for rna-seq data with deseq2. Genome Biol.

[2] Robinson MD, McCarthy DJ, Smyth GK (2010). edger: a bioconductor package for differential expression analysis of digital gene expression data. Bioinformatics.

[3] Law CW, Chen Y, Shi W, Smyth GK (2014). voom: Precision weights unlock linear model analysis tools for rna-seq read counts. Genome Biol.

[4] Zhou Y-H, Xia K, Wright FA (2011). A powerful and flexible approach to the analysis of rna sequence count data. Bioinformatics.

[5] Robinson MD, Smyth GK (2007). Moderated statistical tests for assessing differences in tag abundance. Bioinformatics.

[6] McCarthy DJ, Chen Y, Smyth GK (2012). Differential expression analysis of multifactor rna-seq experiments with respect to biological variation. Nucleic Acids Res.

[7] Chen Y, Lun AT, Smyth GK. Differential expression analysis of complex rna-seq experiments using edger. In: Statistical Analysis of Next Generation Sequencing Data. Springer: 2014. p. 51–74. 10.1007/978-3-319-07212-8_3.

[8] Lund SP, Nettleton D, McCarthy DJ, Smyth GK. Detecting differential expression in rna-sequence data using quasi-likelihood with shrunken dispersion estimates. Stat Appl Genet Mol Biol. 2012; 11(5). 10.1515/1544-6115.1826 23104842

[9] Lun AT, Chen Y, Smyth GK (2016). It’s de-licious: a recipe for differential expression analyses of rna-seq experiments using quasi-likelihood methods in edger. Statistical Genomics.

[10] Smyth GK (2004). Linear models and empirical bayes methods for assessing differential expression in microarray experiments. Stat Appl Genet Mol Biol.

[11] Stricker G, Engelhardt A, Schulz D, Schmid M, Tresch A, Gagneur J (2017). Genogam: genome-wide generalized additive models for chip-seq analysis. Bioinformatics.

[12] Thurston SW, Wand M, Wiencke JK (2000). Negative binomial additive models. Biometrics.

[13] Anders S, Huber W (2010). Differential expression analysis for sequence count data. Genome Biol.

[14] Bozdag S, Li A, Riddick G, Kotliarov Y, Baysan M, Iwamoto FM, Cam MC, Kotliarova S, Fine HA (2013). Age-specific signatures of glioblastoma at the genomic, genetic, and epigenetic levels. PLoS ONE.

[15] Benjamini Y, Hochberg Y (1995). Controlling the false discovery rate: a practical and powerful approach to multiple testing. J R Stat Soc Ser B (Methodol).

[16] Kuan P-F, Waszczuk MA, Kotov R, Clouston S, Yang X, Singh PK, Glenn ST, Gomez EC, Wang J, Bromet E (2017). Gene expression associated with ptsd in world trade center responders: An rna sequencing study. Transl Psychiatry.

[17] Weathers FW, Litz BT, Herman DS, Huska JA, Keane TM, et al.The PTSD checklist (PCL): Reliability, validity, and diagnostic utility. In: Annual Convention of the International Society for Traumatic Stress Studies, San Antonio, TX (Vol. 462). San Antonio: 1993. https://scholar.google.com.sg/scholar?q=the+ptsd+checklist+(pcl)+reliability+validity+ and+diagnostic+utility\&hl=en&as_sdt=0&as_vis=1&oi=scholart.

[18] Kuan PF, Waszczuk M, Kotov R, Marsit C, Guffanti G, Yang X, Koenen K, Bromet E, Luft B (2017). Dna methylation associated with ptsd and depression in world trade center responders: An epigenome-wide study. Biol Psychiatry.

[19] Wood SN, Pya N, Säfken B (2016). Smoothing parameter and model selection for general smooth models. J Am Stat Assoc.

[20] Wood SN. J R Stat Soc Ser B (Stat Methodol). 2011; 73(1):3–36.

[21] Wood SN (2004). Stable and efficient multiple smoothing parameter estimation for generalized additive models. J Am Stat Assoc.

[22] Wood SN (2017). Generalized Additive Models: an Introduction with R.

[23] Wood SN (2003). Thin plate regression splines. J R Stat Soc Ser B (Stat Methodol).

[24] Core Team R. C. T. R.R: A Language and Environment for Statistical Computing. Vienna: R Foundation for Statistical Computing: 2013. https://www.R-project.org/.

[25] Friedman J, Hastie T, Tibshirani R (2001). The Elements of Statistical Learning, (vol. 1, No. 10).

[26] Prautzsch H, Boehm W, Paluszny M. Bézier and B-spline Techniques: Springer Science & Business Media; 2013.

[27] Yu G, Wang L-G, Han Y, He Q-Y (2012). clusterprofiler: an r package for comparing biological themes among gene clusters. Omics: J Integr Biol.

[28] Kanehisa M, Goto S (2000). Kegg: kyoto encyclopedia of genes and genomes. Nucleic Acids Res.

[29] Ashburner M, Ball CA, Blake JA, Botstein D, Butler H, Cherry JM, Davis AP, Dolinski K, Dwight SS, Eppig JT (2000). Gene ontology: tool for the unification of biology. Nat Genet.

[30] Morley J, Baumgartner R (2004). Cytokine-related aging process. J Gerontol Ser A Biol Sci Med Sci.

[31] Rea IM, Gibson DS, McGilligan V, McNerlan SE, Alexander HD, Ross OA (2018). Age and age-related diseases: role of inflammation triggers and cytokines. Front Immunol.

[32] Chung HY, Cesari M, Anton S, Marzetti E, Giovannini S, Seo AY, Carter C, Yu BP, Leeuwenburgh C (2009). Molecular inflammation: underpinnings of aging and age-related diseases. Ageing Res Rev.

[33] Franceschi C, Campisi J (2014). Chronic inflammation (inflammaging) and its potential contribution to age-associated diseases. J Gerontol Ser A Biomed Sci Med Sci.

[34] Reya T, Clevers H (2005). Wnt signalling in stem cells and cancer. Nature.

[35] Gruber J, Yee Z, Tolwinski N (2016). Developmental drift and the role of wnt signaling in aging. Cancers.

[36] Sprenger CC, Plymate SR, Reed MJ (2010). Aging-related alterations in the extracellular matrix modulate the microenvironment and influence tumor progression. Int J Cancer.

[37] Hastie T, Package R. Generalized Additive Models. 2019. R package version, 1(1) http://cran.r-project.org/web/packages/gam/index.html.

[38] Jacob L, Neuvial P, Dudoit S (2012). More power via graph-structured tests for differential expression of gene networks. Ann Appl Stat.

