# Supplementary material for: Negative binomial additive model for RNA-Seq data analysis
Source: BMC Bioinformatics. 2020 May 1;21:171. doi: 10.1186/s12859-020-3506-x (PMC7195715; doi:10.1186/s12859-020-3506-x)
Supplement: Supplementary file 1 — Additional file 1 This file contains supplementary information, figures and tables. [file 12859_2020_3506_MOESM1_ESM.pdf]

Supplementary Materials for Negative Binomial Additive Model for RNA-Seq Data Analysis

Xu Ren and Pei Fen Kuan*

Department of Applied Mathematics and Statistics,
Stony Brook University,
Stony Brook, NY 11794, USA

*Correspondence to peifen.kuan@stonybrook.edu

Information A: Model Specification and Statistical Inference

Generalized Additive Model

For gene i and sample j, we assume the gene count K_{ij} follows the generalized additive model:

$$K_{ij} \sim \text{NB}(\mu_{ij}, \alpha_i), \quad \log(\mu_{ij}) = \log(s_j) + \sum_r f_r(x_{jr})$$

where $\mu_{ij} = \text{E}(K_{ij})$ is the mean count and α_i is dispersion parameter that relates the mean to the variance by $\text{Var}(K_{ij}) = \mu_{ij} + \alpha_i \mu_{ij}^2$, and x_{jr}'s are the r covariates of interest. The logarithm of normalization factor $\log(s_j)$ is an offset in the model and s_j is the normalization factor computed by the median-of-ratios as in DESeq2 for sequencing depth adjustment (see Sequencing Depth Normalization section). $f_r(\cdot)$, $r = 1, 2, ..., R$ represents the spline or smooth function which capture the nonlinear associations between covariate r and gene expression. Since our statistical goal is to conduct inference on genes to ascertain whether they are DE with respect to the covariate/phenotype of interest, we choose $f_r(\cdot)$ to be spline functions to facilitate the inference, i.e., $f_r(x_{jr}) = \sum_{q=1}^{Q} \beta_{rq} B_{rq}(x_{jr})$, where β_{rq} are unknown coefficients, $B(\cdot)$ are B-spline basis functions, and Q is total number of basis functions. The coefficients $\boldsymbol{\beta} = (\beta_{11}, ..., \beta_{1Q}, ..., \beta_{R1}, ..., \beta_{RQ})^T$ are estimated by the penalized log-likelihood maximization to avoid overfitting. In particular, the model is estimated by maximizing

$$L(\boldsymbol{\beta}) = l(\boldsymbol{\beta}) - \frac{1}{2} \sum_r \lambda_r \boldsymbol{\beta}^{\text{T}} \mathbf{S}_r \boldsymbol{\beta} \quad (1)$$

with respect to $\boldsymbol{\beta}$, where $l(\cdot)$ is the log-likelihood function of negative binomial distribution, λ_r is smoothing parameter which controls the smoothness of $f_r(\cdot)$, and $\mathbf{S}_r$ is a positive semidefinite matrix.

Model Fitting

Several methods have been proposed for fitting the generalized additive model. Hastie and Tibshirani (1986, 1990) [1, 2] proposed a general backfitting algorithm, whereas Thurston et al. (2000) [3] adapted the backfitting algorithm for negative binomial distribution. In the backfitting algorithm, given the smoothing parameters λ_r and dispersion parameters α_i, the coefficients $\boldsymbol{\beta}$ can be computed by the penalized iteratively reweighted least squares. However, the most challenging part is in the estimation of the smoothing parameters [4], which is not easy to be incorporated in the backfitting algorithm. Wood (2006, 2011); Wood et al. (2016) [5, 6, 7] proposed a nested iterative algorithm which estimates the smoothing parameters and coefficients simultaneously. Their algorithm estimates the smoothing

parameters using the cross validation framework in the outer iteration and the coefficients using penalized iteratively reweighted least squares in the inner iteration. We adopted the `gam` function from the mgcv package [7, 6, 8, 4, 9] for fitting the generalized additive model on each gene in NBAMSeq. Using the default setting in `gam` function, the knots to construct the basis functions are placed exactly at the data points.

The smoothing parameter tuning criteria can be divided into two types, namely (a) the prediction error based criteria including AIC, cross-validation or generalized cross-validation (GCV), and (b) the likelihood based criteria including maximum likelihood or restricted maximum likelihood (REML) [6]. The likelihood based methods offer some advantageous over the prediction error based methods since they impose a larger penalty on overfitting and exhibit faster convergence of smoothing parameters estimation [10]. In NBAMSeq, we use the REML method. Maximizing the penalized log-likelihood (1) can be viewed as a Bayesian approach with Gaussian prior $\boldsymbol{\beta} \sim N(\mathbf{0}, \mathbf{S}_\lambda^-)$, where $\mathbf{S}_\lambda = \sum \lambda_r \mathbf{S}_r$ and $\mathbf{S}_\lambda^-$ is the inverse or pseudoinverse [11]. Under the Bayesian framework, estimating the smoothing parameters can be viewed as maximizing the log-marginal likelihood [5, 6, 7]

$$V(\boldsymbol{\lambda}) = \log \int f(\boldsymbol{y}|\boldsymbol{\beta}) f(\boldsymbol{\beta}) d\boldsymbol{\beta}. \tag{2}$$

The Laplacian approximation of (2) is given by:

$$V^*(\boldsymbol{\lambda}) \simeq L(\hat{\boldsymbol{\beta}}) + \frac{1}{2}\log|\mathbf{S}_\lambda|_+ - \frac{1}{2}\log|\mathbf{X}^{\mathrm{T}}\mathbf{W}\mathbf{X} + \mathbf{S}_\lambda| + \frac{M}{2}\log(2\pi),$$

where $\hat{\boldsymbol{\beta}}$ is the maximizer of (1), $\mathbf{X}$ is the model matrix of covariates i.e. $\mathbf{X} = [\mathbf{X}^1, \mathbf{X}^2, ..., \mathbf{X}^R]$ with $\mathbf{X}^r_{jk} = B_{rk}(x_{jr})$. $|\mathbf{S}_\lambda|_+$ denotes the product of positive eigenvalues of $\mathbf{S}_\lambda$, M is the number of zero eigenvalues of $\mathbf{S}_\lambda$, and $\mathbf{W}$ is the diagonal weight matrix. For negative binomial distribution with logarithm link and variance given by $\mu_{ij} + \alpha_i\mu_{ij}^2$, the diagonal elements of $\mathbf{W}$ is given by $w_{jj} = \frac{1}{1/\mu_{ij}+\alpha_i}$. Let $\lambda_{i1}, ..., \lambda_{iR}$ denote the smoothing parameters of gene i. The nested iteration implemented in mgcv for model fitting are summarized as Algorithms 1 and 2.

Algorithm 1 Outer iteration for $\boldsymbol{\lambda}$ and α_i

1: Initialize $\boldsymbol{\rho} = (\log \alpha_i, \log \lambda_{i1}, ..., \log \lambda_{iR})$ and $\boldsymbol{\beta}$.
2: Calculate $\hat{\boldsymbol{\beta}}$ by Algorithm 2.
3: Calculate $\frac{\partial V}{\partial \rho_x}$ and $\frac{\partial^2 V}{\partial \rho_x \partial \rho_y}$ for all x, y.
4: Denote gradient by ∇V and the Hessian matrix by H, where $\mathrm{H}_{xy} = \frac{\partial^2 V}{\partial \rho_x \partial \rho_y}$. Calculate $\boldsymbol{\Delta} = -\mathrm{H}^{-1} \nabla V$.
5: If $V(\boldsymbol{\rho} + \boldsymbol{\Delta}) < V(\boldsymbol{\rho})$, set $\boldsymbol{\Delta}$ to be $\boldsymbol{\Delta}/2$ until $V(\boldsymbol{\rho} + \boldsymbol{\Delta}) > V(\boldsymbol{\rho})$.
6: Set $\boldsymbol{\rho}$ to be $\boldsymbol{\rho} + \boldsymbol{\Delta}$.
7: Repeat Step 2 to Step 6 until $\frac{\partial V}{\partial \rho_x} \simeq 0$ for all x and $-\mathrm{H}$ is positive semidefinite.

Algorithm 2 Inner iteration for $\boldsymbol{\beta}$

1: Initialize $\boldsymbol{\beta}$.
2: Update $\boldsymbol{\beta}$ by $\boldsymbol{\beta}^{\text{new}} = (\mathbf{X}^{\mathrm{T}}\mathbf{W}\mathbf{X} + \mathbf{S}_{\lambda})^{-1}\mathbf{X}^{\mathrm{T}}\mathbf{W}\mathbf{z}$, where $\mathbf{z}$ is a vector with each element $z_j = \log(\mu_{ij}/s_j) + (K_{ij} - \mu_{ij})/\mu_{ij}$.
3: Update $\mathbf{W}$ by $w_{jj} = \frac{1}{1/\mu_{ij} + \alpha_i}$.
4: Repeat Step 2 and Step 3 until the change of $\boldsymbol{\beta}$ below threshold.

Algorithm 3 Workflow of NBAMSeq

1: Estimate normalization factors s_j.
2: Estimate smoothing parameters and gene-wise dispersions by Algorithms 1 and 2.
3: Estimate dispersion trend and prior variance using gene-wise dispersion estimates from Step 2.
4: Calculate MAP estimate of dispersions.
5: Estimate coefficients $\boldsymbol{\beta}$ by algorithm 2 using the smoothing parameters in Step 2 and dispersion parameters in Step 4.

Dispersion Estimation

Information sharing across genes in the estimation of dispersion parameter has been shown to increase accuracy compared to treating each gene independently, especially when the number of replicates is small. Numerous methods have been proposed to incorporate information sharing, and most of them were based on the empirical Bayesian approach which shrinks the gene-wise dispersion estimates toward a common dispersion parameter across all genes [12]. Specifically, for each individual gene i, we assume a log-normal prior for α_i:

$$\log(\alpha_i) \sim N(\log \alpha_{\text{tr}}(\bar{\mu}_i), \sigma_d^2).$$

where dispersion trend relates dispersion to mean of normalized $\bar{\mu}_i = \frac{1}{m}\sum_j \frac{K_{ij}}{s_j}$ counts by $\alpha_{\text{tr}}(\bar{\mu}_i) = \frac{b_1}{\bar{\mu}_i} + b_0$ and σ_d^2 is the prior variance, which is assumed to be constant across all genes. A Gamma regression is fitted to estimate the parameters b_1 and b_0. The prior variance σ_d^2 is estimated by subtracting the expected sampling variance of log-dispersion estimator from the variance estimate of residual $\log(\hat{\alpha}_i) - \log \alpha_{\text{tr}}(\bar{\mu}_i)$ as described in Love et al. (2014) [12].

We first obtain gene-wise dispersion estimates $\hat{\alpha}_i$ by Algorithms 1 and 2, which are then used to fit the dispersion trend and estimate the variance in prior distribution. The final dispersion parameter is estimated by maximizing

$$l_{\text{CR}}(\alpha) - \frac{(\log \alpha - \log \alpha_{\text{tr}}(\bar{\mu}_i))^2}{2\sigma_d^2}$$

where l_{CR} is Cox-Reid adjusted log-likelihood [13], which can be viewed as a maximum a posteriori (MAP) approach. The workflow implemented in NBAMSeq is summarized in Algorithm 3.

Inference

Our main objective is to identify the genes that exhibit either linear or nonlinear association with the covariate/phenotype of interest. We formulate this as a hypothesis test $H_0 : f_r(x_r) = 0$ versus $H_1 : f_r(x_r) \neq 0$, where $f_r(\cdot)$ is a smooth function of the covariate of interest. Since we use a Bayesian framework in the coefficients estimation, we have $f(\boldsymbol{\beta}|\boldsymbol{y}) \propto f(\boldsymbol{y}|\boldsymbol{\beta})f(\boldsymbol{\beta})$. In addition, $\boldsymbol{\beta} \sim N(\mathbf{0}, \mathbf{S}_\lambda^-)$, thus $\log f(\boldsymbol{\beta}|\boldsymbol{y}) \propto \log f(\boldsymbol{y}|\boldsymbol{\beta}) - \frac{1}{2}\boldsymbol{\beta}^{\text{T}}\mathbf{S}_\lambda\boldsymbol{\beta}$. By applying a second order Taylor expansion to $\hat{\boldsymbol{\beta}}$, we obtain an approximation

$$\log f(\boldsymbol{y}|\hat{\boldsymbol{\beta}}) - \frac{1}{2}\hat{\boldsymbol{\beta}}^{\text{T}}\mathbf{S}_\lambda\hat{\boldsymbol{\beta}} + \frac{1}{2}(\boldsymbol{\beta} - \hat{\boldsymbol{\beta}})^{\text{T}}(\text{D}^2 \log f(\boldsymbol{y}|\hat{\boldsymbol{\beta}}) - \mathbf{S}_\lambda)(\boldsymbol{\beta} - \hat{\boldsymbol{\beta}}) \quad (3)$$

where $\mathrm{D}^2 \log f(\boldsymbol{y}|\hat{\boldsymbol{\beta}})$ is Hessian matrix of the log-likelihood of negative binomial distribution with respect to $\boldsymbol{\beta}$. We use the expected information matrix to replace the Hessian matrix (see Expected Hessian Matrix section) and equation (3) simplifies to

$$\log f(\boldsymbol{y}|\hat{\boldsymbol{\beta}}) - \frac{1}{2}\hat{\boldsymbol{\beta}}^{\mathrm{T}}\mathbf{S}_{\lambda}\hat{\boldsymbol{\beta}} - \frac{1}{2}(\boldsymbol{\beta} - \hat{\boldsymbol{\beta}})^{\mathrm{T}}(\mathbf{X}^{\mathrm{T}}\mathbf{W}\mathbf{X} + \mathbf{S}_{\lambda})(\boldsymbol{\beta} - \hat{\boldsymbol{\beta}}) \tag{4}$$

The large sample limit of (4) is a multivariate normal distribution, thus $\boldsymbol{\beta}|\boldsymbol{y} \sim N(\hat{\boldsymbol{\beta}}, (\mathbf{X}^{\mathrm{T}}\mathbf{W}\mathbf{X} + \mathbf{S}_{\lambda})^{-1})$ and $\mathbf{V}_{\boldsymbol{\beta}} = (\mathbf{X}^{\mathrm{T}}\mathbf{W}\mathbf{X} + \mathbf{S}_{\lambda})^{-1}$ is Bayesian covariance matrix of $\boldsymbol{\beta}$. Let $\mathbf{f}_r$ be the values of $f_r(x_r)$ evaluated at the observed values of x_r and $\tilde{\mathbf{X}}$ denote the matrix such that $\mathbf{f}_r = \tilde{\mathbf{X}}\boldsymbol{\beta}$. Wood (2012) [14] showed that $\hat{\mathbf{f}}_r \sim N(\mathbf{f}_r, \tilde{\mathbf{X}}\mathbf{V}_{\boldsymbol{\beta}}\tilde{\mathbf{X}}^{\mathrm{T}})$ approximately and under the null hypothesis the test statistics $\hat{\mathbf{f}}_r^{\mathrm{T}}(\tilde{\mathbf{X}}\mathbf{V}_{\boldsymbol{\beta}}\tilde{\mathbf{X}}^{\mathrm{T}})^{-1}\hat{\mathbf{f}}_r$ follows a χ^2 distribution with the degrees of freedom (df) equals to the effective degrees of freedom (edf) corresponding to variable x_r if the edf is an integer. If the edf of x_r is not an integer, Wood (2012) [14] showed that the distribution of $\hat{\mathbf{f}}_r^{\mathrm{T}}(\tilde{\mathbf{X}}\mathbf{V}_{\boldsymbol{\beta}}\tilde{\mathbf{X}}^{\mathrm{T}})^{-1}\hat{\mathbf{f}}_r$ can be approximated by a non-central χ^2 distribution using the methods of Liu et al. (2009) [15]. Specifically, the df and the non-central parameter in χ^2 distribution are determined so that the skewness of $\hat{\mathbf{f}}_r^{\mathrm{T}}(\tilde{\mathbf{X}}\mathbf{V}_{\boldsymbol{\beta}}\tilde{\mathbf{X}}^{\mathrm{T}})^{-1}\hat{\mathbf{f}}_r$ and target χ^2 distribution are equal, whereas the difference in kurtosis is minimized. We utilize this result to calculate a p-value for each gene. The p-values are further adjusted using FDR procedure to account for multiple hypothesis testing.

Sequencing Depth Normalization

For RNA-Seq data, the raw counts of each sample are proportional to its sequencing depth i.e. the total number of reads. The raw counts are not directly comparable across samples and therefore normalization factors are needed to adjust for gene counts. Several methods have been developed for RNA-Seq data normalization including total count, upper quartile [16], median, quantile [17, 18], conditional quantile [19], reads per kilobase per million mapped reads (RPKM) [20], median-of-ratios [21], and trimmed mean of M values [22]. A comprehensive comparisons of these normalization methods are provided in Dillies et al. [23] and Li et al. [24]. By comparing the spearman correlation between normalized counts and quantitative reverse transcription polymerase chain reaction (qRT-PCR), Li et al. [24] showed that RPKM yields the best normalization results when alignment accuracy is low. However, by considering the effect of normalization method on DE analysis, Dillies et al. [23] showed that RPKM is ineffective and not recommended. They also showed that the median-of-ratios [21] and trimmed mean of M values [22] maintain low false-positive rate without compromising power in DE analysis.

Throughout this paper, we adapt the median-of-ratios used by DESeq [21], DEXSeq [25]

and DESeq2 [12] for sequencing depth adjustment. Let K_{ij} be the read count for gene i in sample j. The normalization factor of sample j is defined as:

$$s_j = \underset{i:K_i^* \neq 0}{\text{median}} \frac{K_{ij}}{K_i^*}, \text{ where } K_i^* = \Big(\prod_{j=1}^{m} K_{ij}\Big)^{1/m} \tag{5}$$

where m is the total number of samples.

Expected Hessian Matrix

The likelihood function of negative binomial distribution is given by:

$$f(\boldsymbol{\beta}|\boldsymbol{y}) = \prod_{j=1}^{m} \frac{\Gamma(y_j + 1/\alpha)}{\Gamma(y_j)\Gamma(1/\alpha)} \left(\frac{\alpha\mu_j}{1+\alpha\mu_j}\right)^{y_j} \left(\frac{1}{1+\alpha\mu_j}\right)^{1/\alpha}.$$

Thus we have

$$\begin{aligned}
\log f(\boldsymbol{\beta}|\boldsymbol{y}) &= \sum_{j=1}^{m} \left[y_j \log \frac{\alpha\mu_j}{1+\alpha\mu_j} + \frac{1}{\alpha} \log \frac{1}{1+\alpha\mu_j} + h(y_j) \right] \\
&= \sum_{j=1}^{m} \Big[y_j \log\alpha + y_j \log\mu_j - (y_j + 1/\alpha)\log(1+\alpha\mu_j) + h(y_j) \Big] \\
&= \sum_{j=1}^{m} \Big[y_j \log\alpha + y_j (\textstyle\sum \beta_r x_{jr}) - (y_j + 1/\alpha)\log(1+\alpha\mu_j) + h(y_j) \Big].
\end{aligned}$$

Taking the partial derivative with respect to β, we have

$$\begin{aligned}
\frac{\partial \log f(\boldsymbol{\beta}|\boldsymbol{y})}{\partial \beta_a} &= \sum_{j=1}^{m} \left[y_j x_{ja} - (y_j + 1/\alpha) \frac{\alpha}{1+\alpha\mu_j} \frac{\partial \mu_j}{\partial \beta_a} \right] \\
&= \sum_{j=1}^{m} \left[y_j x_{ja} - (y_j + 1/\alpha) \frac{\alpha\mu_j}{1+\alpha\mu_j} x_{ja} \right] \\
&= \sum_{j=1}^{m} \left[y_j x_{ja} - (y_j + 1/\alpha) x_{ja} + (y_j + 1/\alpha) \frac{1}{1+\alpha\mu_j} x_{ja} \right],
\end{aligned}$$

$$\begin{aligned}
\frac{\partial^2 \log f(\boldsymbol{\beta}|\boldsymbol{y})}{\partial \beta_a \partial \beta_b} &= -\sum_{j=1}^{m} \left[(y_j + 1/\alpha) x_{ja} \frac{\alpha}{(1+\alpha\mu_j)^2} \frac{\partial \mu_j}{\partial \beta_b} \right] \\
&= -\sum_{j=1}^{m} \left[(y_j + 1/\alpha) \frac{\alpha\mu_j}{(1+\alpha\mu_j)^2} x_{ja} x_{jb} \right].
\end{aligned} \tag{6}$$

We take the expectation of (6) and obtain

$$E\left[\frac{\partial^2 \log f(\boldsymbol{\beta}|\boldsymbol{y})}{\partial\beta_a\partial\beta_b}\right] = -\sum_{j=1}^{m}\left[(\mu_j + 1/\alpha)\frac{\alpha\mu_j}{(1+\alpha\mu_j)^2}x_{ja}x_{jb}\right] = -\sum_{j=1}^{m}\left[\frac{\mu_j}{1+\alpha\mu_j}x_{ja}x_{jb}\right]. \tag{7}$$

Equation (7) is precisely the a, b element of $-\mathbf{X}^{\mathrm{T}}\mathbf{W}\mathbf{X}$, where $\mathbf{W}$ is the diagonal weight matrix with diagonal elements $w_{jj} = \frac{1}{1/\mu_j+\alpha}$.

Table S1

Table S1: ANOVA F-test summary.

comparison	test statistics	degrees of freedom	number of significant genes
(i) vs (ii)	$(\frac{\text{SSE}_1-\text{SSE}_2}{df_1-df_2})/(\frac{\text{SSE}_2}{df_2})$	1,154	464
(i) vs (iii)	$(\frac{\text{SSE}_1-\text{SSE}_3}{df_1-df_3})/(\frac{\text{SSE}_3}{df_3})$	6,149	250
(ii) vs (iii)	$(\frac{\text{SSE}_2-\text{SSE}_3}{df_2-df_3})/(\frac{\text{SSE}_3}{df_3})$	5,149	9

Table S2

As discussed in the main manuscript (Motivating datasets section), 250 genes are significant when comparing model (i) to model (iii), among which 30 genes have FDR greater than 0.2 when comparing model (i) to model (ii). Table S2 shows the p-value and FDR of these 30 genes, which suggests that they are nonlinear age specific gene signature for GBM as they cannot be detected by linear regression.

Table S2: List of the 30 genes with FDR greater than 0.2.

gene	p-value	FDR	gene	p-value	FDR
AGGF1	0.2633	0.5884	LOC647946	0.9190	0.9730
ARL13B	0.0622	0.3127	MIPEP	0.6125	0.8330
ARNTL	0.0383	0.2452	NBN	0.2066	0.5357
ATP10D	0.3525	0.6684	NEDD4	0.5251	0.7854
CNOT2	0.5770	0.8142	NFE2L2	0.0274	0.2086
COL11A2	0.0391	0.2472	OS9	0.9172	0.9722
CPSF6	0.3188	0.6420	PRDM11	0.1179	0.4174
ETAA1	0.6731	0.8637	RAB3IP	0.2630	0.5883
GEFT	0.0919	0.3729	RTP1	0.9207	0.9737
GPI	0.0802	0.3521	SKIL	0.1008	0.3904
HNF4G	0.1174	0.4167	TAF8	0.0328	0.2270
KCNMB4	0.6686	0.8620	TMEM147	0.1868	0.5136
KIAA0040	0.0259	0.2028	TNFRSF13C	0.0257	0.2026
LOC145837	0.1093	0.4050	YEATS4	0.9026	0.9670
LOC153684	0.0971	0.3826	ZSCAN2	0.0253	0.2014

Table S3

To illustrate that the nonlinear age effect is prevalent among other types of cancer, we analyzed other TCGA RNA-Seq data downloaded from the Broad GDAC Firehose database.

The gene filtering criterion, library size normalization method and hypothesis tests are similar to the framework discussed in the main manuscript (Motivating datasets section). Samples with missing age, race or gender information are excluded from the analysis. A total of 25 cancer types are analyzed. Table S3 shows the sample size, number of genes after filtering, number of significant genes under raw p-value 0.05 and FDR 0.05 of comparing model (i) vs. ii), (i) vs. (iii), and (ii) vs. (iii).

Table S3: Summary of Broad GDAC Firehose database analysis.

Cancer	sample size	number of genes	i) vs. ii) p-value	i) vs. ii) FDR	i) vs. iii) p-value	i) vs. iii) FDR	ii) vs. iii) p-value	ii) vs. iii) FDR
ACC	68	15065	1091	0	1113	4	1012	3
BLCA	391	16989	3111	245	1462	0	716	0
BRCA	983	17405	7437	5707	5647	3257	1691	17
CHOL	36	14862	600	0	1033	1	1100	1
COAD	258	16006	1317	2	898	0	757	0
DLBC	48	13983	521	0	439	2	381	2
ESCA	164	16652	3551	589	2651	174	1350	20
GBM	158	16089	2859	464	2351	250	1381	9
HNSC	505	16902	2140	50	1199	1	751	1
KICH	64	14740	472	0	261	1	250	1
KIRC	525	16756	2876	268	1171	12	407	0
KIRP	273	16365	3243	477	2310	81	1351	3
LAML	171	15291	3524	835	2003	119	794	10
LGG	504	16540	7840	6554	6165	4487	1618	0
LIHC	357	16305	5012	2635	2434	338	447	0
LUAD	437	17226	4236	1236	2229	9	727	0
LUSC	387	17223	3090	295	2148	28	1217	2
MESO	87	15454	695	0	631	0	651	0
PAAD	174	16450	3036	36	692	0	168	0
PCPG	175	15857	2258	175	1750	20	1163	0
SARC	249	16700	6252	4045	5620	2768	2725	365
SKCM	451	16826	4374	1398	3219	277	1483	1
STAD	356	17252	4682	1652	3269	93	1233	3
THCA	410	16030	3527	1025	2119	201	818	0
THYM	117	15882	6314	4079	3196	51	628	0

We further investigated common age specific gene expression signature across all cancer types. When comparing model (i) to model (ii), 15 genes (AIMP2, ANKRD6, CNTN1, DLC1, FAM64A, GPR160, GREB1L, MGA, MST1R, MYBL2, NEFH, PDE1A, RANBP17, TSPYL5, ZNF518B) are significant under raw p-value 0.05 in at least 13 cancer types. When comparing model (i) to model (iii), 25 genes are significant under raw p-value 0.05 in at least 10 cancer types. However, no gene is significant across all cancer types at raw p-value 0.05,

suggesting that aging gene expression signatures are cancer specific. A more detailed study of age-rated gene signature is discussed in Yang et al. [26].

Table S4

Table S4: Correct classification percentage (3 classes).

	m=15*	m=20	m=25	m=30	m=35	m=40
edf	0.9716	0.9752	0.9781	0.9804	0.9814	0.9828
AIC	0.9706	0.9739	0.9765	0.9789	0.9796	0.9808
BIC	0.9706	0.9739	0.9765	0.9789	0.9797	0.9808

*m stands for sample size.

Table S5

Table S5: Correct classification percentage (2 classes).

	m=15*	m=20	m=25	m=30	m=35	m=40
edf	0.8295	0.8412	0.8499	0.8606	0.8689	0.8726
AIC	0.7905	0.7994	0.8028	0.8174	0.8210	0.8203
BIC	0.7910	0.7996	0.8035	0.8178	0.8224	0.8221

*m stands for sample size.

Table S6

Table S6: Significant gene sets selected by DESeq2 (GBM dataset).

Ontology	ID	Description	pvalue	FDR
BP	GO:0003002	regionalization	1.8170E-07	0.0007
BP	GO:0061053	somite development	6.9090E-07	0.0009
BP	GO:0009952	anterior/posterior pattern specification	8.5141E-07	0.0009
BP	GO:0007389	pattern specification process	9.2558E-07	0.0009
BP	GO:0072001	renal system development	1.0208E-05	0.0083
BP	GO:0001763	morphogenesis of a branching structure	1.6160E-05	0.0108
BP	GO:0071772	response to BMP	2.1659E-05	0.0108
BP	GO:0071773	cellular response to BMP stimulus	2.1659E-05	0.0108
BP	GO:0030509	BMP signaling pathway	2.6860E-05	0.0108
BP	GO:0090092	regulation of transmembrane receptor protein serine/threonine kinase signaling pathway	2.8865E-05	0.0108
BP	GO:0001822	kidney development	2.9261E-05	0.0108
BP	GO:0001655	urogenital system development	3.3420E-05	0.0114
BP	GO:0007498	mesoderm development	4.3995E-05	0.0138
BP	GO:0001708	cell fate specification	7.0672E-05	0.0206
BP	GO:0016570	histone modification	9.8991E-05	0.0239
BP	GO:0030326	embryonic limb morphogenesis	1.0189E-04	0.0239
BP	GO:0035113	embryonic appendage morphogenesis	1.0189E-04	0.0239
BP	GO:0090100	positive regulation of transmembrane receptor protein serine/threonine kinase signaling pathway	1.0976E-04	0.0239
BP	GO:0001942	hair follicle development	1.1263E-04	0.0239
BP	GO:0007178	transmembrane receptor protein serine/threonine kinase signaling pathway	1.1714E-04	0.0239
BP	GO:0022404	molting cycle process	1.4063E-04	0.0249
BP	GO:0022405	hair cycle process	1.4063E-04	0.0249
BP	GO:0098773	skin epidermis development	1.4063E-04	0.0249
BP	GO:0001756	somitogenesis	1.8889E-04	0.0304
BP	GO:0061138	morphogenesis of a branching epithelium	1.8937E-04	0.0304
BP	GO:0060021	palate development	1.9370E-04	0.0304
BP	GO:0060541	respiratory system development	2.0365E-04	0.0308
BP	GO:0035282	segmentation	2.6298E-04	0.0383
BP	GO:0042303	molting cycle	3.1600E-04	0.0401
BP	GO:0042633	hair cycle	3.1600E-04	0.0401
BP	GO:0001657	ureteric bud development	3.2003E-04	0.0401
BP	GO:0048736	appendage development	3.2481E-04	0.0401
BP	GO:0060173	limb development	3.2481E-04	0.0401
BP	GO:0072163	mesonephric epithelium development	3.5227E-04	0.0410
BP	GO:0072164	mesonephric tubule development	3.5227E-04	0.0410
BP	GO:0001658	branching involved in ureteric bud morphogenesis	3.7316E-04	0.0422
BP	GO:0035239	tube morphogenesis	3.9142E-04	0.0422
BP	GO:0016575	histone deacetylation	4.2111E-04	0.0422
BP	GO:0010463	mesenchymal cell proliferation	4.2425E-04	0.0422
BP	GO:0010862	positive regulation of pathway-restricted SMAD protein phosphorylation	4.2425E-04	0.0422
BP	GO:0060393	regulation of pathway-restricted SMAD protein phosphorylation	4.2438E-04	0.0422
BP	GO:0035137	hindlimb morphogenesis	4.3555E-04	0.0423
BP	GO:0030510	regulation of BMP signaling pathway	4.6576E-04	0.0442
BP	GO:0001823	mesonephros development	5.1014E-04	0.0453
BP	GO:0035107	appendage morphogenesis	5.1132E-04	0.0453
BP	GO:0035108	limb morphogenesis	5.1132E-04	0.0453

Table S7

Table S7: Significant gene sets selected by NBAMSeq (GBM dataset).

Ontology	ID	Description	pvalue	FDR
CC	GO:0005578	proteinaceous extracellular matrix	1.58E-07	8.05E-05
BP	GO:0009952	anterior/posterior pattern specification	6.72E-06	0.0091
BP	GO:0003002	regionalization	7.54E-06	0.0091
BP	GO:0090092	regulation of transmembrane receptor protein serine/threonine kinase signaling pathway	9.66E-06	0.0091
BP	GO:0090100	positive regulation of transmembrane receptor protein serine/threonine kinase signaling pathway	9.81E-06	0.0091
BP	GO:0042303	molting cycle	1.32E-05	0.0091
BP	GO:0042633	hair cycle	1.32E-05	0.0091
BP	GO:0061053	somite development	1.41E-05	0.0091
BP	GO:0071772	response to BMP	3.35E-05	0.0168
BP	GO:0071773	cellular response to BMP stimulus	3.35E-05	0.0168
BP	GO:0072001	renal system development	5.43E-05	0.0245
BP	GO:0001655	urogenital system development	6.20E-05	0.0252
BP	GO:0001942	hair follicle development	7.33E-05	0.0252
BP	GO:0030510	regulation of BMP signaling pathway	7.33E-05	0.0252
BP	GO:0030509	BMP signaling pathway	7.82E-05	0.0252
BP	GO:0022404	molting cycle process	9.72E-05	0.0253
BP	GO:0022405	hair cycle process	9.72E-05	0.0253
BP	GO:0098773	skin epidermis development	9.72E-05	0.0253
BP	GO:0030198	extracellular matrix organization	1.01E-04	0.0253
BP	GO:0043062	extracellular structure organization	1.08E-04	0.0254
BP	GO:0030111	regulation of Wnt signaling pathway	1.13E-04	0.0254
BP	GO:0007389	pattern specification process	1.24E-04	0.0266
BP	GO:0001763	morphogenesis of a branching structure	1.43E-04	0.0293
BP	GO:0042634	regulation of hair cycle	1.58E-04	0.0309
BP	GO:0007178	transmembrane receptor protein serine/threonine kinase signaling pathway	1.96E-04	0.0357
BP	GO:0001822	kidney development	1.98E-04	0.0357
BP	GO:0035239	tube morphogenesis	2.12E-04	0.0359
BP	GO:0007369	gastrulation	2.15E-04	0.0359

Table S8

Table S8: Significant gene sets selected by edgeR (GBM dataset).

Ontology	ID	Description	pvalue	FDR
BP	GO:0061053	somite development	2.14E-06	0.0086
BP	GO:0009952	anterior/posterior pattern specification	4.62E-06	0.0092
BP	GO:0003002	regionalization	1.27E-05	0.0170
BP	GO:0007389	pattern specification process	3.22E-05	0.0322
BP	GO:0010862	positive regulation of pathway-restricted SMAD protein phosphorylation	4.94E-05	0.0395
MF	GO:0032549	ribonucleoside binding	2.18E-05	0.0101
MF	GO:0001882	nucleoside binding	3.10E-05	0.0101
MF	GO:0005525	GTP binding	8.95E-05	0.0116
MF	GO:0032561	guanyl ribonucleotide binding	9.10E-05	0.0116
MF	GO:0019001	guanyl nucleotide binding	9.53E-05	0.0116
MF	GO:0032550	purine ribonucleoside binding	1.08E-04	0.0116
MF	GO:0001883	purine nucleoside binding	1.24E-04	0.0116

Table S9

Table S9: Significant gene sets among DE genes unique to NBAMSeq (GBM dataset).

Ontology	ID	Description	pvalue	FDR
CC	GO:0005578	proteinaceous extracellular matrix	1.58E-07	8.05E-05
BP	GO:0030198	extracellular matrix organization	1.01E-04	0.02531
BP	GO:0043062	extracellular structure organization	1.08E-04	0.02540
BP	GO:0042634	regulation of hair cycle	1.58E-04	0.03091
BP	GO:0007369	gastrulation	2.15E-04	0.03590

Table S10

Table S10: Significant KEGG pathways found by NBAMSeq (WTC dataset).

ID	Description	pvalue	FDR
hsa05016	Huntington disease	7.15E-07	1.91E-04
hsa04330	Notch signaling pathway	1.79E-04	0.01730
hsa05322	Systemic lupus erythematosus	1.94E-04	0.01730
hsa05169	Epstein-Barr virus infection	3.00E-04	0.01999
hsa00190	Oxidative phosphorylation	6.42E-04	0.03117
hsa04722	Neurotrophin signaling pathway	7.00E-04	0.03117
hsa05203	Viral carcinogenesis	8.20E-04	0.03129
hsa01522	Endocrine resistance	1.43E-03	0.04488
hsa05014	Amyotrophic lateral sclerosis (ALS)	1.51E-03	0.04488

Figure S1

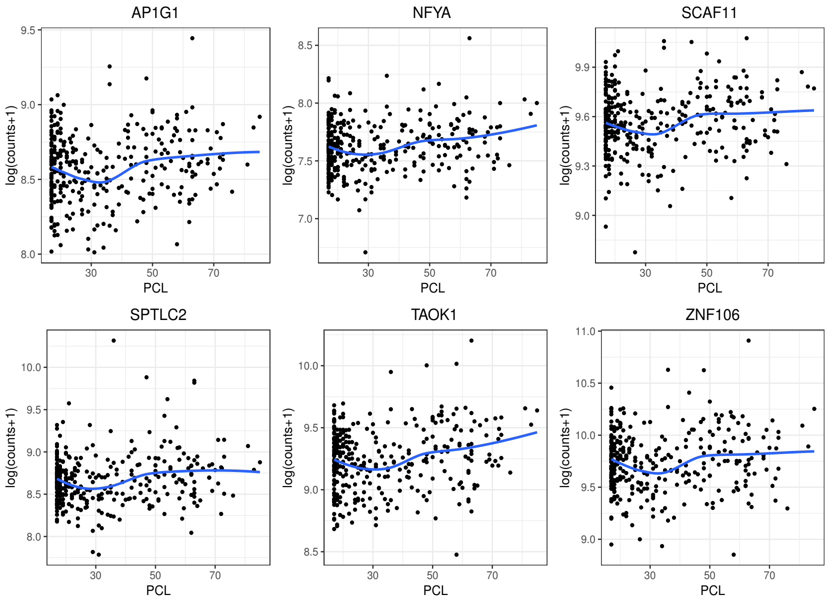

Figure S1: Nonlinear relationship between gene counts and PCL.

Figure S2 and S3

It is undeniable that some DE analysis methods may be sensitive to the proportion of DE genes. We tested the sensitivity of our proposed method by considering different proportions of DE genes. We considered the simulation scenario I with median dispersion ($a = 3$) in the main manuscript (Simulation section). The simulation settings were the same as discussed in the main manuscript except that we randomly selected 10% and 20% genes to be differentially expressed. Figure S2 shows that the dispersion estimates obtained by NBAMSeq remains accurate regardless of the DE proportion. The number of DE genes, TPR, AUC and F1 score are given in Figure S3, which show that NBAMSeq still achieves the best performance among all methods. Moreover, NBAMSeq controls FDR well with empirical FDR < 0.03 across all DE proportions. These results illustrate that NBAMSeq is not sensitive to the proportion of DE genes.

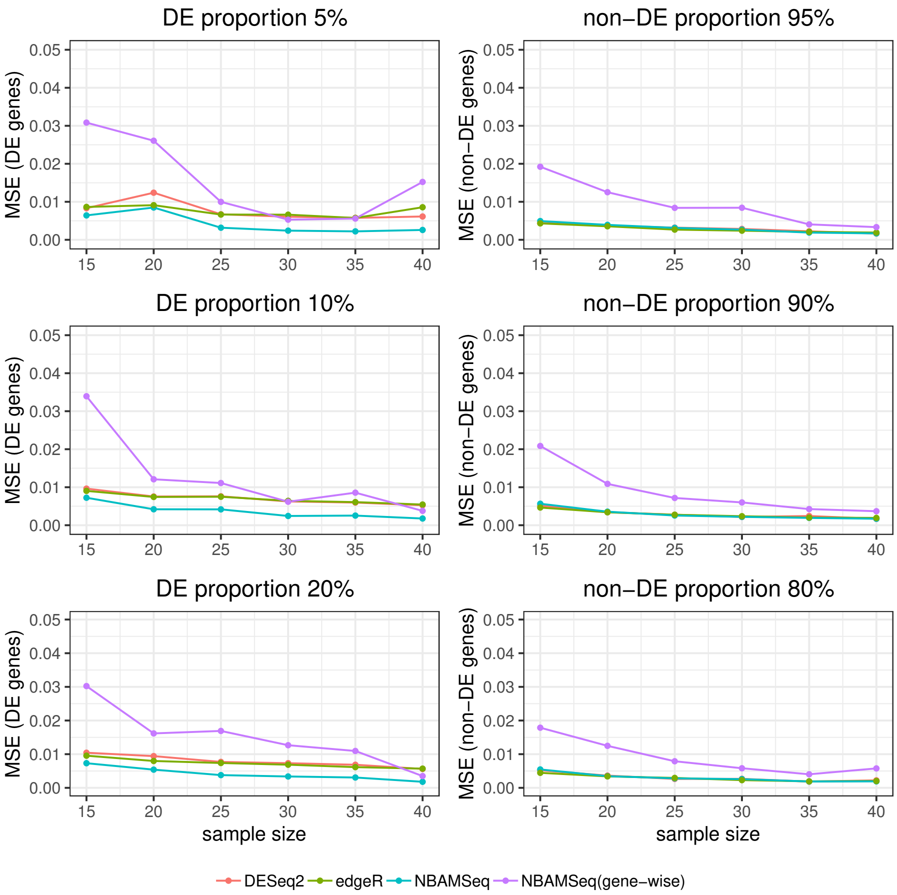

Figure S2: MSE of dispersion estimates under different DE proportion.

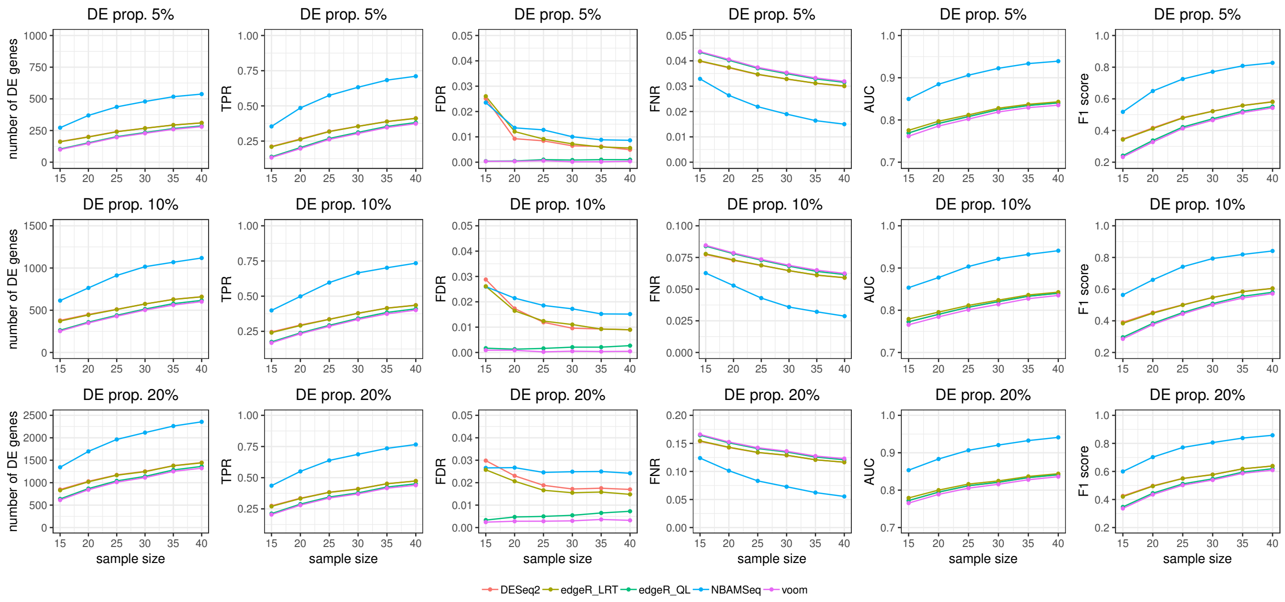

Figure S3: Performance metrics under different DE proportion.

Figure S4 and S5

As mentioned in the main manuscript (Simulation section), Lund et al [27] showed that QL approach controls FDR better than LRT approach when detecting DE genes. Here we investigated the performance of NBAMSeq using QL approach. Following Lund et al [27], when detecting DE genes, we compared the test statistic:

$$\mathrm{F}_{\mathrm{QL}} = \frac{\mathrm{LR}_i/q_i}{\hat{\Phi}_i}$$

to an F distribution with q_i and $\hat{d} + m - p_i$ degrees of freedom, where LR_i is twice the difference between the likelihood of null and full model in likelihood ratio test, q_i is the difference of the effective degree of freedom, p_i is the effective degree of freedom in the full model, m is the sample size; $\hat{d}$ and $\hat{\Phi}_i$ are estimated using Smyth's [28] method of moment approach. Besides the NBAMSeq QL approach, another two methods are also included in the comparison, namely the Gaussian additive model (GAM) and ANOVA F-test. In GAM, we modeled the logarithm transformed of read count (log(count+1)) of each gene by a Gaussian distribution. We used `gam` function with Gaussian family in mgcv package [7, 6, 8, 4, 9] to fit a GAM model for each gene and the DE test was conducted using the `summary` function. For the test statistics and degrees of freedom, see Wood [4] for details. The ANOVA F-test is similar to our analysis in the motivating dataset of the main manuscript with the exception

that only model (i) vs (iii) were considered. Raw p-values for testing model (i) vs (iii) were calculated and then adjusted using Benjamini &Hochberg [29] FDR. For both GAM and ANOVA, genes with FDR < 0.05 were declared as DE genes. In addition, as discussed in the main manuscript Implementation section, we set the `gamma` argument in `gam` function to 2.5 instead of using the default value of 1. Here we investigate the performance of NBAMSeq if `gamma=1` is used (denoted by NBAMSeq1). Our simulation setup is identical to the setup described in the main manuscript. The results are shown in Figure S4 (nonlinear scenario) and Figure S5 (linear scenario).

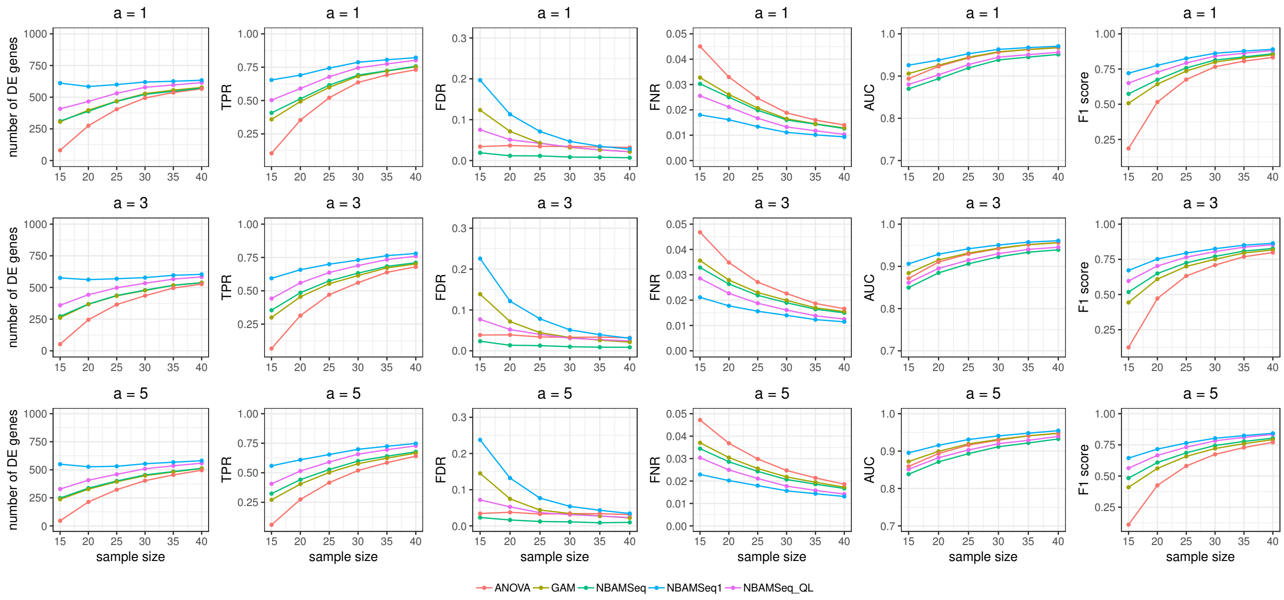

Figure S4: Performance metrics in Scenario I (other methods).

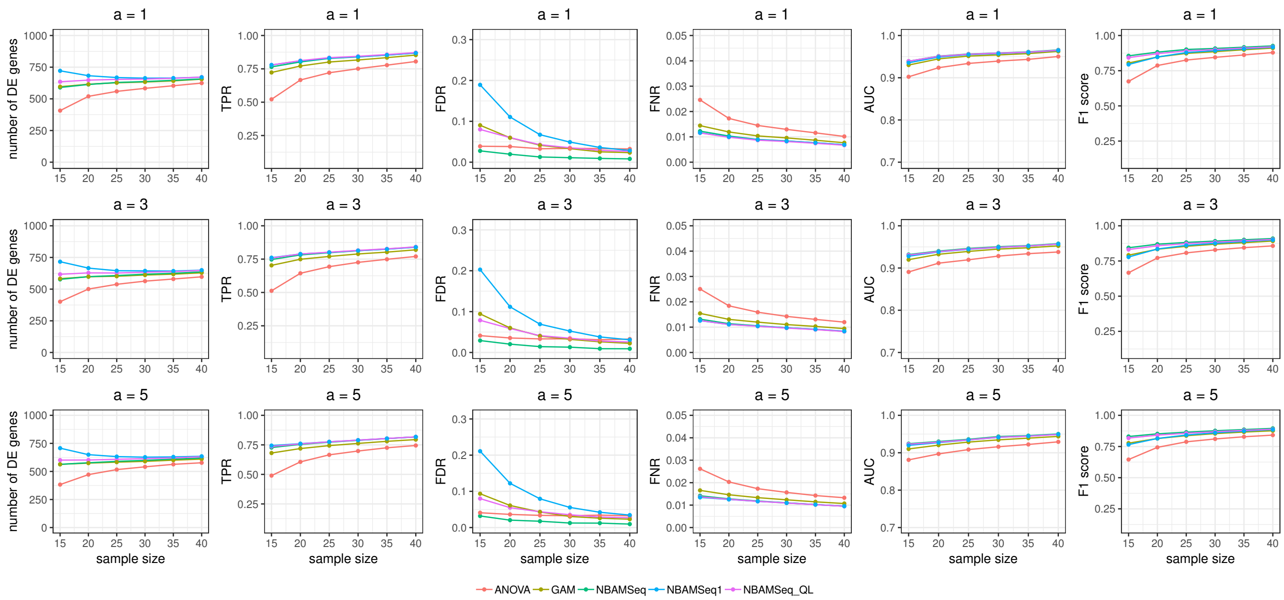

Figure S5: Performance metrics in Scenario II (other methods).

Although NBAMSeq QL approach yields high TPR and low FNR compared to other methods, it is unable to control the FDR especially when the sample size is fewer than 20, where its empirical FDR is inflated. GAM has comparable TPR but higher FDR compared to NBAMSeq, which indicates that modeling RNASeq count data by Gaussian distribution results in loss of power and inflated false discoveries. ANOVA controls FDR all cases, however its TPR and AUC is lowest among all the methods, implying that it is not powerful in detecting differentially expressed genes. NBAMSeq with the default `gamma=1` in `gam` function yields inflated FDR in most cases. Thus we set `gamma=2.5` by default to enforce a larger penalty when fitting the generalized additive model. Our proposed method NBAMSeq with `gamma=2.5` yields a higher power while controlling FDR, indicating that it has the best performance compared to other methods.

Figure S6

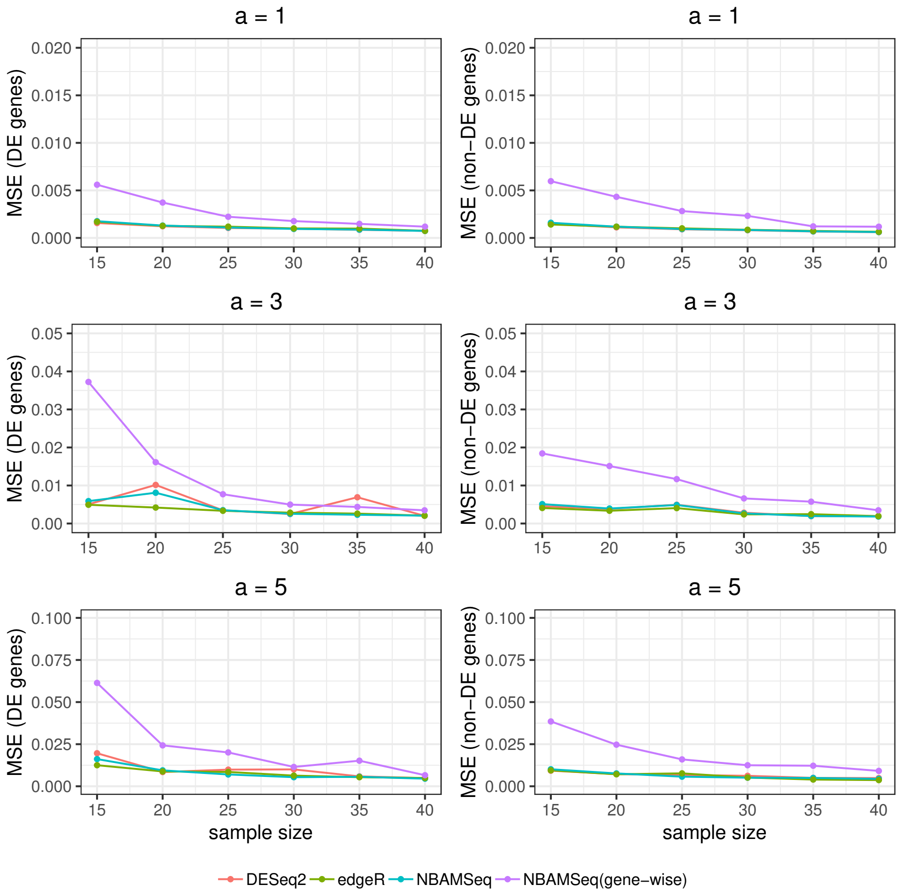

Figure S6: Scenario II: MSE of dispersion estimates.

Figure S7

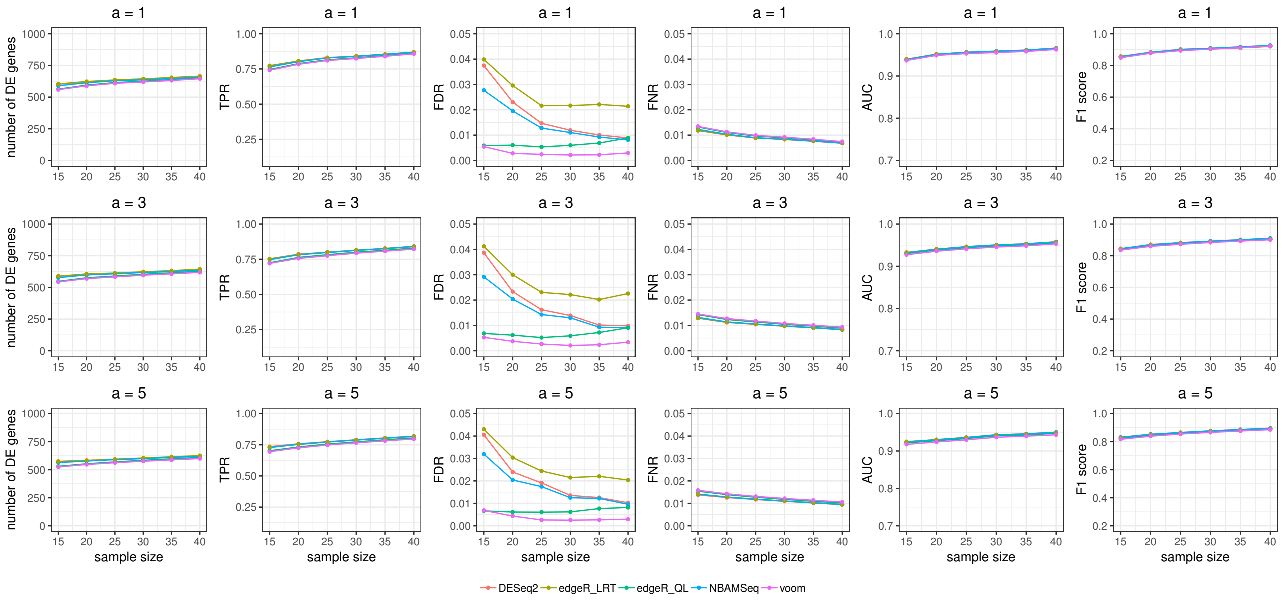

Figure S7: Performance metrics in Scenario II.

Figure S8

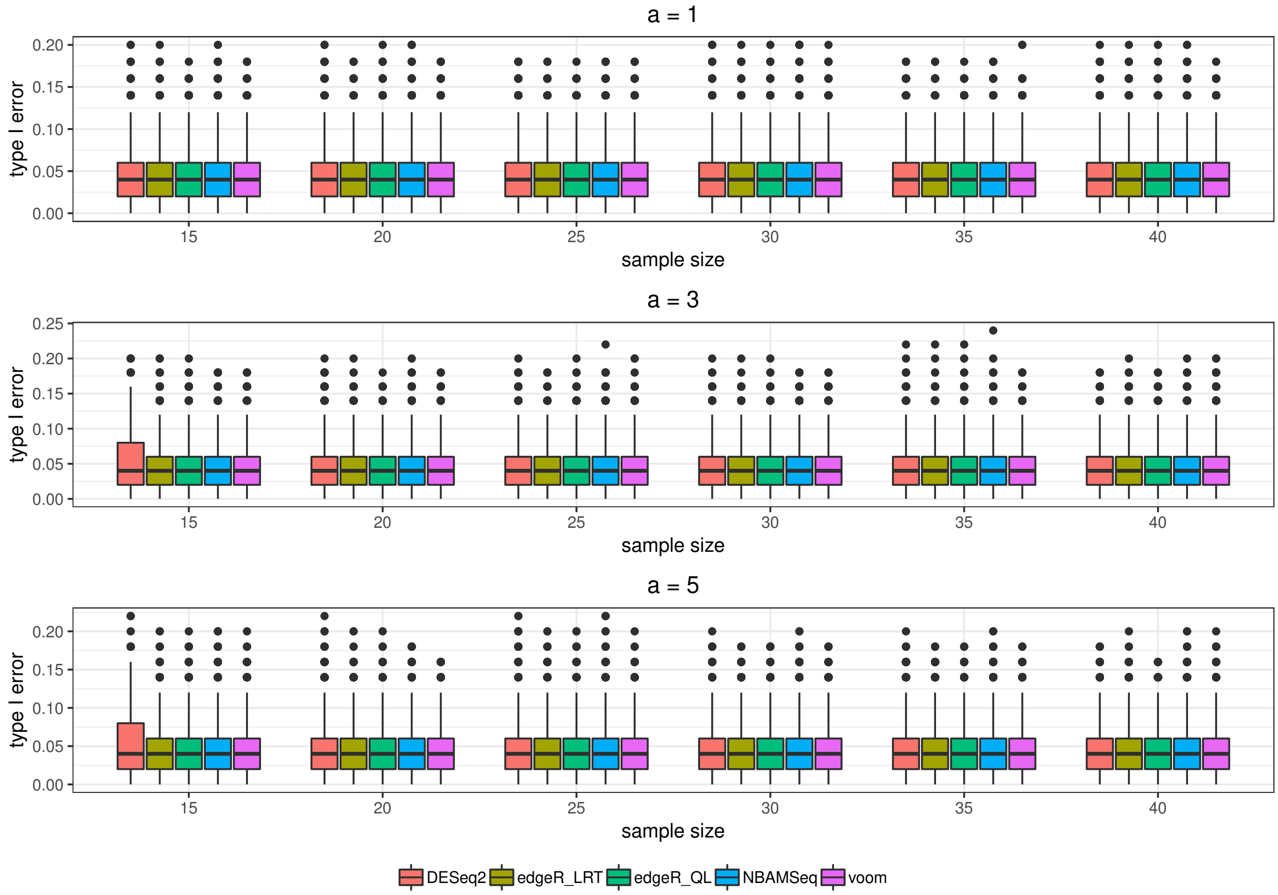

Figure S8: Scenario III: type I error of DESeq2, edgeR and NBAMSeq.

Figure S9

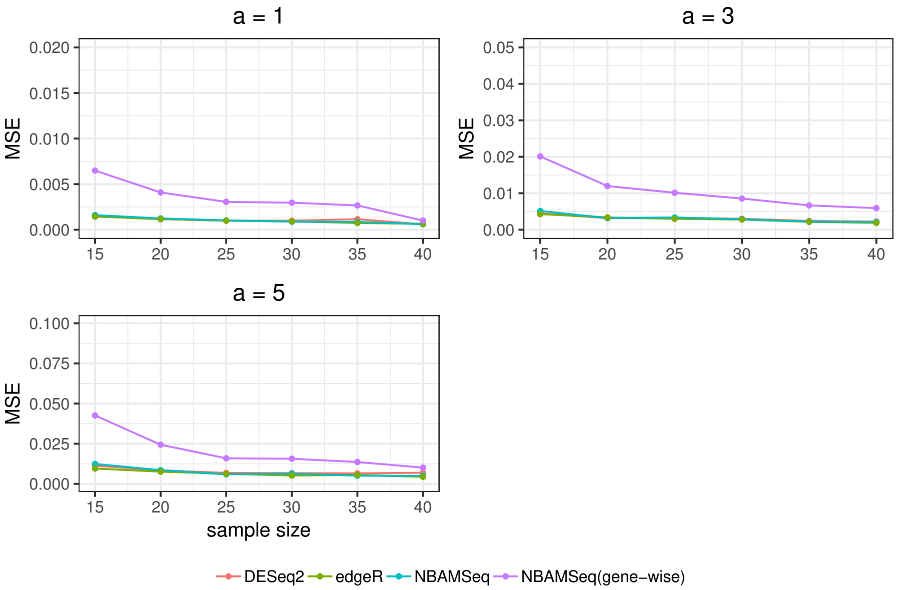

Figure S9: Scenario III: MSE of dispersion estimates.

Figure S10

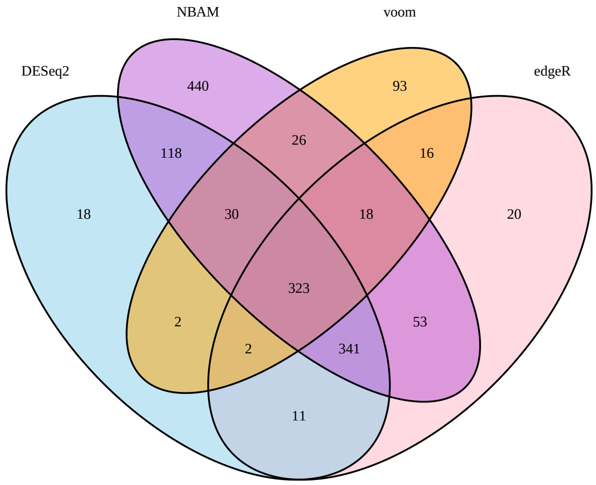

Figure S10: Number of significant genes by DESeq2, edgeR and NBAMSeq (GBM dataset).

Figure S11

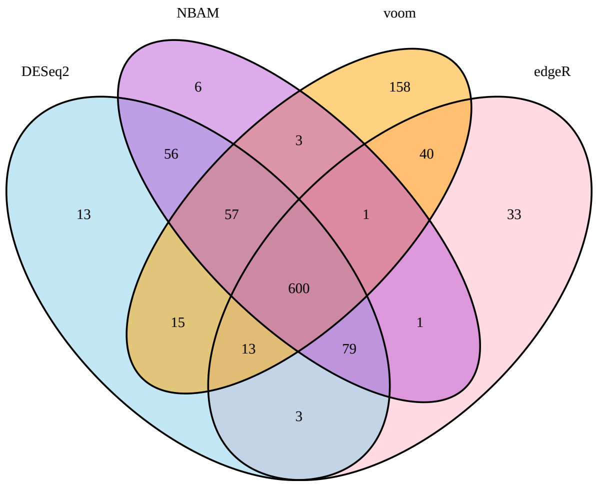

Figure S11: Number of significant genes by DESeq2, edgeR, and NBAMSeq (WTC dataset).

Figure S12

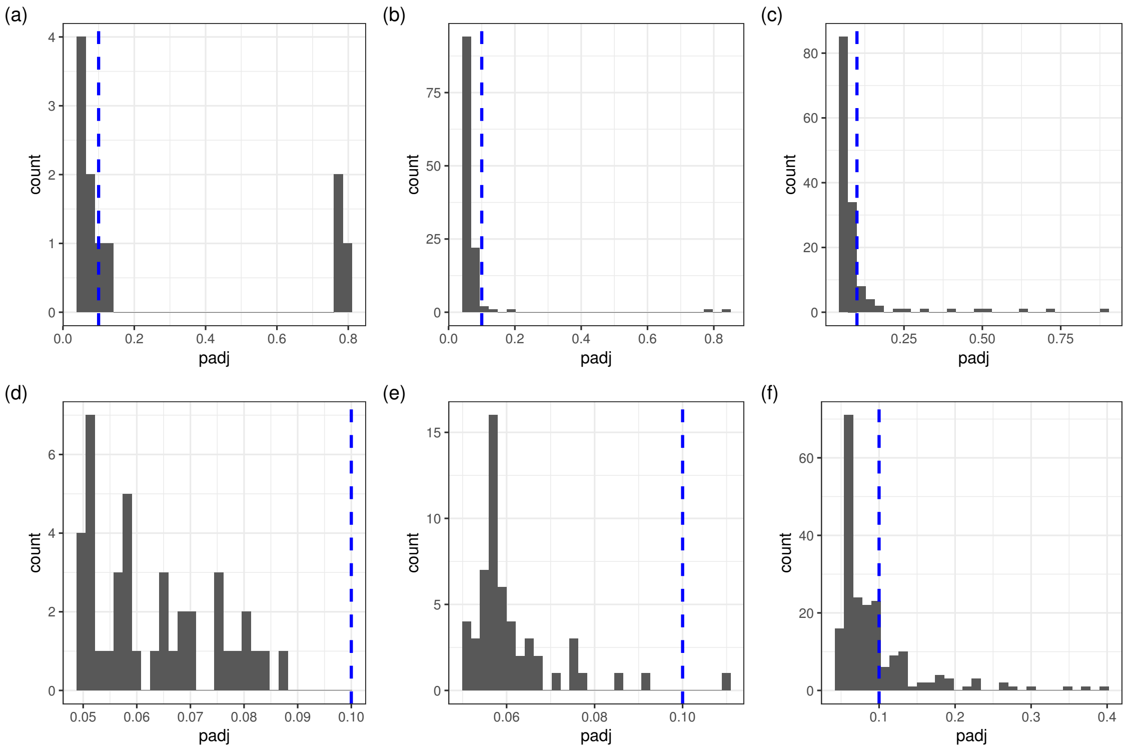

Figure S12: Histogram of adjusted p-values (WTC dataset). The dashed line is FDR cut-off 0.1. (a) Genes detected by NBAMSeq but not DESeq2. (b) Genes detected by NBAMSeq but not edgeR. (c) Genes detected by NBAMSeq but not voom. (d) Genes detected by DESeq2 but not NBAMSeq. (e) Genes detected by edgeR but not NBAMSeq. (f) Genes detected by voom but not NBAMSeq.

Figure S13 - S15

An alternative approach for detecting the nonlinear association between phenotype of interest and gene expression is via DESeq2, edgeR or voom with the design matrix generated by the basis of spline regression. However, this approach requires the degrees of freedom (df) in the spline regression to be fixed, and may result in DE analysis that is sensitive to the choice of df. On the other hand, NBAMSeq does not require the df to be fixed and the df is estimated within the algorithm automatically. Without loss of generality, we illustrate this advantage of NBAMSeq using the simulation with median dispersion ($a = 3$) in the main manuscript (Simulation section). For DESeq2 and edgeR, we considered the scenario where the true underlying relationship between gene counts and covariate is nonlinear. We first fixed the df

and generated the B-spline basis matrix for polynomial spline. This basis matrix was used to construct the design matrix in DESeq2 and edgeR. The accuracy of dispersion estimates and FDR in DE tests are given in Figure S13 and S14, which shows their FDRs are inflated if the df is misspecified, especially when sample size is small. For voom, we considered both the linear and nonlinear scenarios for the underlying true association. The design matrix was constructed in the same way as DESeq2 and edgeR. The TPR and FNR are given in Figure S15, which show that voom with fixed df is less powerful compared to NBAMSeq as given by higher FNRs.

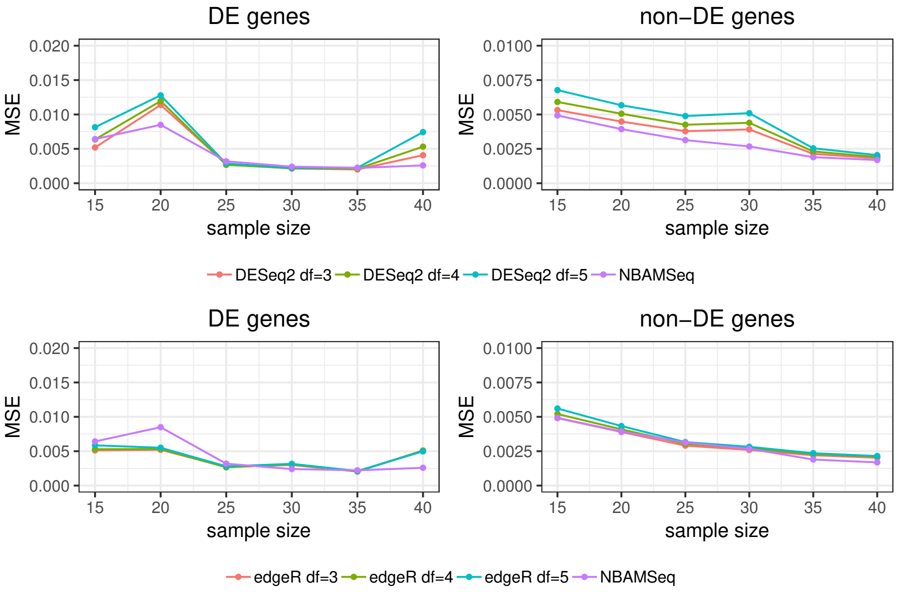

Figure S13: MSE of dispersion estimates given by DESeq2 and edgeR with fixed df.

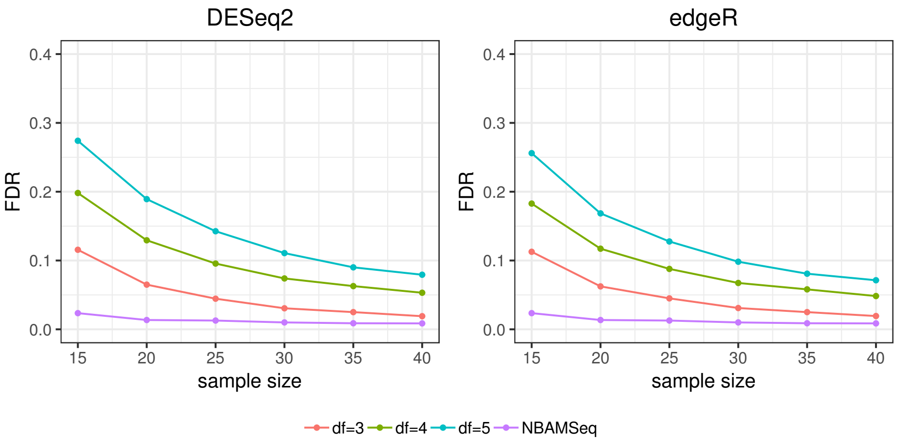

Figure S14: FDR of DESeq2 and edgeR with fixed df.

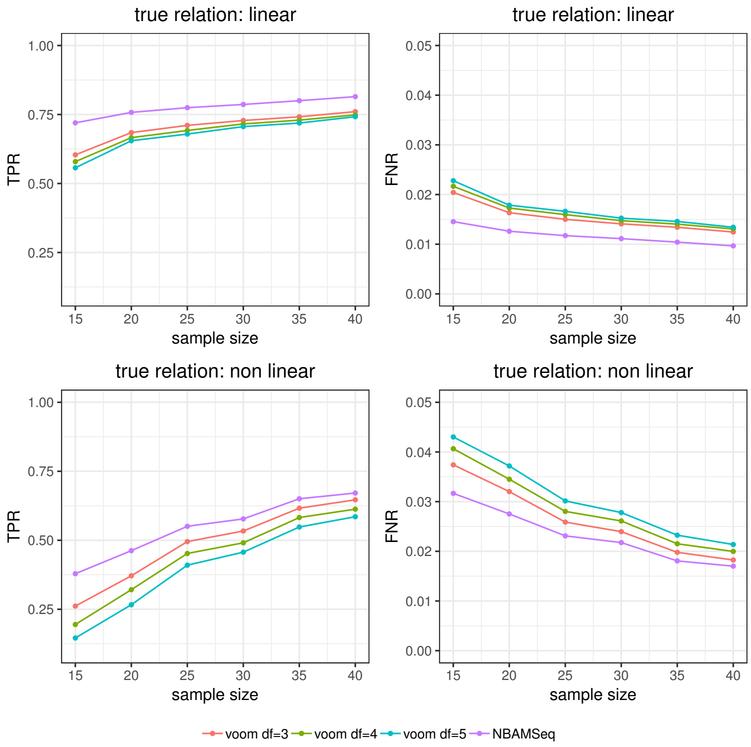

Figure S15: TPR and FNR of voom with fixed df.

References

[1] Trevor Hastie and Robert Tibshirani. Generalized additive models. *Statist. Sci.*, 1(3):297–310, 08 1986.

[2] TJ Hastie and RJ Tibshirani. *Generalized additive models. Chapman & Hall: CRC Monographs on Statistics & Applied Probability.* London, 1990.

[3] Sally W Thurston, MP Wand, and John K Wiencke. Negative binomial additive models. *Biometrics*, 56(1):139–144, 2000.

[4] Simon N Wood. *Generalized additive models: an introduction with R.* CRC press, 2017.

[5] Simon N Wood. *Generalized additive models: an introduction with R.* Chapman and Hall/CRC, 2006.

[6] Simon N Wood. Fast stable restricted maximum likelihood and marginal likelihood estimation of semiparametric generalized linear models. *Journal of the Royal Statistical Society: Series B (Statistical Methodology)*, 73(1):3–36, 2011.

[7] Simon N Wood, Natalya Pya, and Benjamin Säfken. Smoothing parameter and model selection for general smooth models. *Journal of the American Statistical Association*, 111(516):1548–1563, 2016.

[8] Simon N Wood. Stable and efficient multiple smoothing parameter estimation for generalized additive models. *Journal of the American Statistical Association*, 99(467):673–686, 2004.

[9] Simon N Wood. Thin plate regression splines. *Journal of the Royal Statistical Society: Series B (Statistical Methodology)*, 65(1):95–114, 2003.

[10] Wolfgang Härdle, Peter Hall, and James Stephen Marron. How far are automatically chosen regression smoothing parameters from their optimum? *Journal of the American Statistical Association*, 83(401):86–95, 1988.

[11] Adi Ben-Israel and Thomas NE Greville. *Generalized inverses: theory and applications*, volume 15. Springer Science & Business Media, 2003.

[12] Michael I Love, Wolfgang Huber, and Simon Anders. Moderated estimation of fold change and dispersion for rna-seq data with deseq2. *Genome biology*, 15(12):550, 2014.

[13] David Roxbee Cox and Nancy Reid. Parameter orthogonality and approximate conditional inference. *Journal of the Royal Statistical Society. Series B (Methodological)*, pages 1–39, 1987.

[14] Simon N Wood. On p-values for smooth components of an extended generalized additive model. *Biometrika*, 100(1):221–228, 2012.

[15] Huan Liu, Yongqiang Tang, and Hao Helen Zhang. A new chi-square approximation to the distribution of non-negative definite quadratic forms in non-central normal variables. *Computational Statistics & Data Analysis*, 53(4):853–856, 2009.

[16] James H Bullard, Elizabeth Purdom, Kasper D Hansen, and Sandrine Dudoit. Evaluation of statistical methods for normalization and differential expression in mrna-seq experiments. *BMC bioinformatics*, 11(1):94, 2010.

[17] Benjamin M Bolstad, Rafael A Irizarry, Magnus Åstrand, and Terence P. Speed. A comparison of normalization methods for high density oligonucleotide array data based on variance and bias. *Bioinformatics*, 19(2):185–193, 2003.

[18] Yee Hwa Yang and Natalie P Thorne. Normalization for two-color cdna microarray data. *Lecture Notes-Monograph Series*, pages 403–418, 2003.

[19] Kasper D Hansen, Rafael A Irizarry, and Zhijin Wu. Removing technical variability in rna-seq data using conditional quantile normalization. *Biostatistics*, 13(2):204–216, 2012.

[20] Ali Mortazavi, Brian A Williams, Kenneth McCue, Lorian Schaeffer, and Barbara Wold. Mapping and quantifying mammalian transcriptomes by rna-seq. *Nature methods*, 5(7):621, 2008.

[21] Simon Anders and Wolfgang Huber. Differential expression analysis for sequence count data. *Genome biology*, 11(10):R106, 2010.

[22] Mark D Robinson and Alicia Oshlack. A scaling normalization method for differential expression analysis of rna-seq data. *Genome biology*, 11(3):R25, 2010.

[23] Marie-Agnès Dillies, Andrea Rau, Julie Aubert, Christelle Hennequet-Antier, Marine Jeanmougin, Nicolas Servant, Céline Keime, Guillemette Marot, David Castel, Jordi Estelle, et al. A comprehensive evaluation of normalization methods for illumina high-throughput rna sequencing data analysis. *Briefings in bioinformatics*, 14(6):671–683, 2013.

[24] Peipei Li, Yongjun Piao, Ho Sun Shon, and Keun Ho Ryu. Comparing the normalization methods for the differential analysis of illumina high-throughput rna-seq data. *BMC bioinformatics*, 16(1):347, 2015.

[25] Simon Anders, Alejandro Reyes, and Wolfgang Huber. Detecting differential usage of exons from rna-seq data. *Genome research*, 22(10):2008–2017, 2012.

[26] Jialiang Yang, Tao Huang, Francesca Petralia, Quan Long, Bin Zhang, Carmen Argmann, Yong Zhao, Charles V Mobbs, Eric E Schadt, Jun Zhu, et al. Synchronized age-related gene expression changes across multiple tissues in human and the link to complex diseases. *Scientific reports*, 5:15145, 2015.

[27] Steven P Lund, Dan Nettleton, Davis J McCarthy, and Gordon K Smyth. Detecting differential expression in rna-sequence data using quasi-likelihood with shrunken dispersion estimates. *Statistical applications in genetics and molecular biology*, 11(5), 2012.

[28] Gordon K Smyth. Linear models and empirical bayes methods for assessing differential expression in microarray experiments. *Statistical applications in genetics and molecular biology*, 3(1):1–25, 2004.

[29] Yoav Benjamini and Yosef Hochberg. Controlling the false discovery rate: a practical and powerful approach to multiple testing. *Journal of the royal statistical society. Series B (Methodological)*, pages 289–300, 1995.
